# A comprehensive satellite-based assessment across the Pacific Arctic Distributed Biological Observatory shows widespread late-season sea surface warming and sea ice declines with significant influences on primary productivity

**DOI:** 10.1371/journal.pone.0287960

**Published:** 2023-07-11

**Authors:** Karen E. Frey, Josefino C. Comiso, Larry V. Stock, Luisa N. C. Young, Lee W. Cooper, Jacqueline M. Grebmeier

**Affiliations:** 1 Graduate School of Geography, Clark University, Worcester, Massachusetts, United States of America; 2 Cryospheric Sciences Laboratory, NASA Goddard Space Flight Center, Greenbelt, Maryland, United States of America; 3 Chesapeake Biological Laboratory, University of Maryland Center for Environmental Science, Solomons, Maryland, United States of America; University of Copenhagen, DENMARK

## Abstract

Massive declines in sea ice cover and widespread warming seawaters across the Pacific Arctic region over the past several decades have resulted in profound shifts in marine ecosystems that have cascaded throughout all trophic levels. The Distributed Biological Observatory (DBO) provides sampling infrastructure for a latitudinal gradient of biological “hotspot” regions across the Pacific Arctic region, with eight sites spanning the northern Bering, Chukchi, and Beaufort Seas. The purpose of this study is two-fold: (a) to provide an assessment of satellite-based environmental variables for the eight DBO sites (including sea surface temperature (SST), sea ice concentration, annual sea ice persistence and the timing of sea ice breakup/formation, chlorophyll-*a* concentrations, primary productivity, and photosynthetically available radiation (PAR)) as well as their trends across the 2003–2020 time period; and (b) to assess the importance of sea ice presence/open water for influencing primary productivity across the region and for the eight DBO sites in particular. While we observe significant trends in SST, sea ice, and chlorophyll-*a*/primary productivity throughout the year, the most significant and synoptic trends for the DBO sites have been those during late summer and autumn (warming SST during October/November, later shifts in the timing of sea ice formation, and increases in chlorophyll-*a*/primary productivity during August/September). Those DBO sites where significant increases in annual primary productivity over the 2003–2020 time period have been observed include DBO1 in the Bering Sea (37.7 g C/m^2^/year/decade), DBO3 in the Chukchi Sea (48.0 g C/m^2^/year/decade), and DBO8 in the Beaufort Sea (38.8 g C/m^2^/year/decade). The length of the open water season explains the variance of annual primary productivity most strongly for sites DBO3 (74%), DBO4 in the Chukchi Sea (79%), and DBO6 in the Beaufort Sea (78%), with DBO3 influenced most strongly with each day of additional increased open water (3.8 g C/m^2^/year per day). These synoptic satellite-based observations across the suite of DBO sites will provide the legacy groundwork necessary to track additional and inevitable future physical and biological change across the region in response to ongoing climate warming.

## Introduction

Arctic sea ice cover is undergoing extensive changes. There has been a pronounced decrease in summer sea ice extent [1], an overall thinning of the ice [2], a lengthening of the melt season [3], and a fundamental shift to a primarily seasonal sea ice cover [4]. Some of the greatest changes in sea ice cover across the pan-Arctic have been observed in the Chukchi and Beaufort seas surrounding Alaska, where there has been substantial secular loss of multi-year ice during summer [5,6]. In contrast, sea ice in the Bering Sea has experienced multi-year patterns of both increases and decreases over the satellite record since the late 1970s in response to broad-scale patterns of atmospheric circulation, with significant increases in winter and spring ice cover observed across the region as recently as 2003–2010 [5]. However, more recent years have brought unprecedented declines in sea ice across the Bering Sea [7–9], including the region south of St. Lawrence Island that in winter 2017–2018 experienced only ~20 days of sea ice cover compared to the long-term average of 149 days [9]. These reductions in sea ice have additionally been found to have important linkages with warming seawaters throughout the region as well [10,11].

The Pacific Arctic sector is also among the most biologically productive marine ecosystems in the world [12–14] and acts as an important sink and possible seasonal source of organic material. Primary productivity across these ecosystems can take the form of open water phytoplankton [15–17] (including deep chlorophyll maxima [18]), under sea ice phytoplankton [19–21], sea ice algae [22,23], and marine macroalgae [24,25]. Although sea ice is a dominant feature on continental shelves at high-latitudes, we are only beginning to understand how shortened seasonal duration of sea ice cover, thinning sea ice, or shifts from multiyear to first-year sea ice (with influences on light, seawater temperature, salinity, and nutrient availability) will specifically affect ecosystems in these regions. In particular, earlier ice-free conditions in spring have disrupted the phenology, species distribution, and abundance of ice algae, water column phytoplankton, and lower trophic level pelagic species [26], and consequently, underlying benthic systems as well as higher trophic level species that are both ultimately reliant upon this water column production [27–30]. This disruption is also characterized by new and/or more abundant phytoplankton species emerging in this rapidly changing environment, where they naturally thrive in a warmer, brighter ocean, including the proliferation of harmful algal [31,32] and under-ice [19,33,34] blooms throughout the region. Future projections of primary production across the Arctic Ocean seem to ubiquitously predict spatial heterogeneity in changes, which can be dependent on several potentially confounding factors. For instance, while some Arctic shelves may have significant increases in primary production with further sea ice declines, the deep central basin of the Arctic Ocean may see only small increases in production owing to low nutrient concentrations; areas that lose ice cover may see decreases in production owing to increased stratification with atmospheric warming; and some inner coastal shelves may see little increase in production owing to the enhanced turbidity from river runoff and coastal erosion [35].

**Fig 1:**
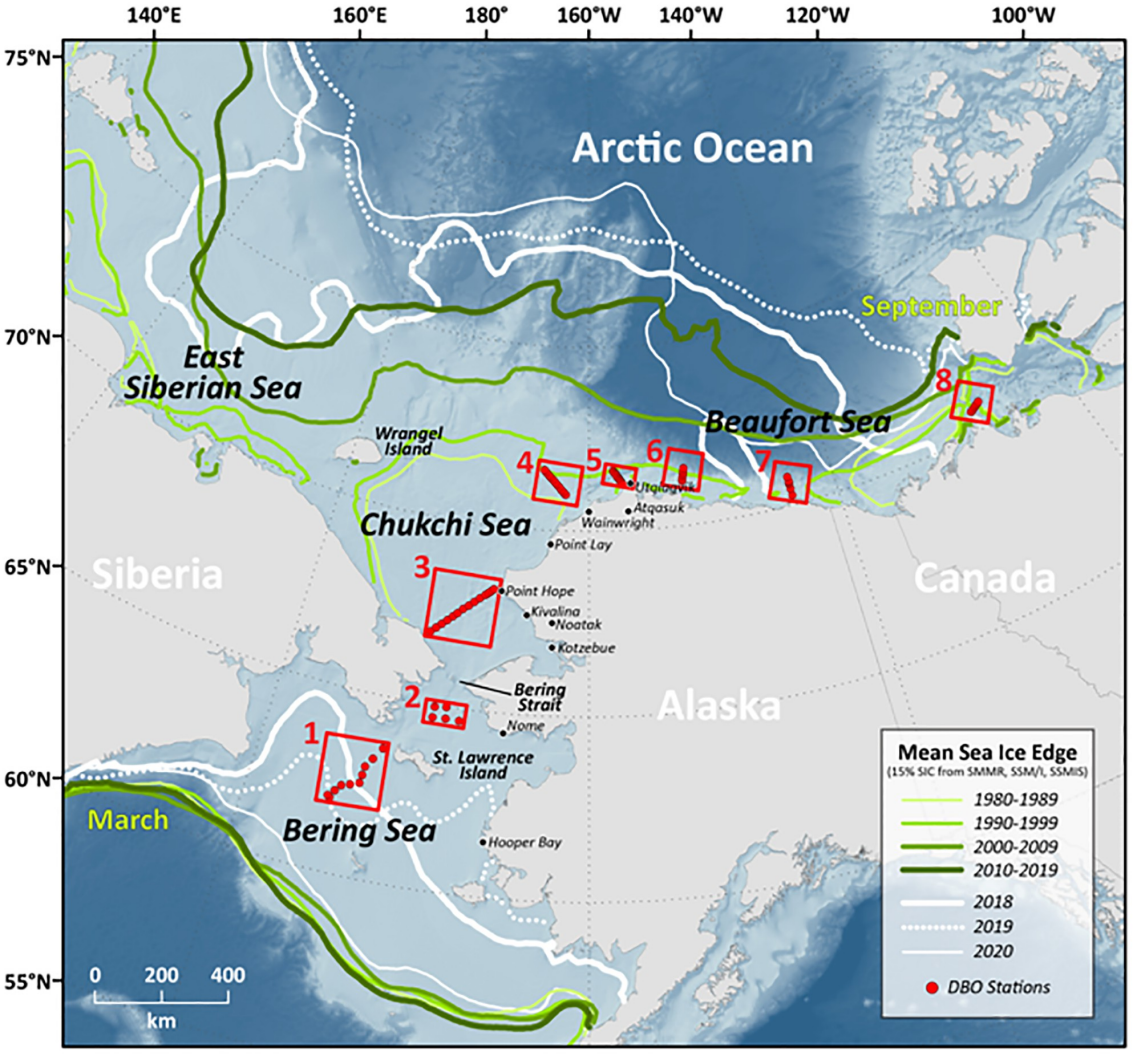
Study area of the eight Distributed Biological Observatory (DBO) sites across the Pacific Arctic region as they relate to the mean sea ice edge, determined with a 15% sea ice concentration threshold using SMMR, SSM/I, and SSMIS satellite data (decadal means as well as the last three years individually: 2018, 2019, and 2020). All sea ice edge contours north (south) of Bering Strait represent September (March) conditions. Basemap datasets from Natural Earth [49] and ESRI [50].

In 2009, it was proposed to develop a Distributed Biological Observatory (DBO) across the Pacific Arctic sector to monitor shifting marine ecosystems in response to recent dramatic sea ice declines and seawater warming in the region [36]. As a result, a DBO field-based pilot program was initiated in 2010, with international participation coordinated by the Pacific Arctic Group (PAG) and US participation managed by NOAA [37]. Since that time, the Pacific Arctic DBO has continued to strengthen with its international coordination and has resulted in journal special issues and hundreds of scientific publications. In particular, the DBO now provides a model sampling infrastructure for a latitudinal gradient of eight biological “hotspot” regions across the Pacific Arctic region (Fig 1). These observations are particularly important given the extensive environmental changes observed throughout the region over the past few decades. Numerous recent studies have illustrated the importance of climate warming for ecosystem dynamics throughout the DBO sites [38–43]. While there have been recent satellite-based studies documenting changes in sea ice cover, sea surface temperature, and primary productivity across the Pacific Arctic region and pan-Arctic as a whole [17,44–48], to date no study has yet provided a synoptic assessment of satellite-based environmental variables for the full suite of these important DBO sites. As such, the purpose of this study is two-fold: (a) to provide an assessment of the variability of satellite-based environmental parameters for the eight DBO sites (including sea surface temperature (SST), sea ice concentration, annual sea ice persistence and the timing of sea ice breakup/formation, chlorophyll-*a* concentrations, primary productivity, and photosynthetically available radiation (PAR)) as well as trends across the 2003–2020 time period; and (b) to assess the impact of sea ice presence in winter and the length of open water in spring, summer, and autumn on primary productivity across the region and for the eight DBO sites in particular.

## Materials and methods

### Satellite sea surface temperature

SST is traditionally measured by ships that regularly navigate the global oceans and also by buoys, moorings and other oceanographic platforms. The data are routinely compiled by the National Oceanic and Atmospheric Administration (NOAA) and time series of SST maps using the data are widely available (e.g. [51]). Because of harsh environmental conditions, there is a paucity of data in polar oceans and the only way to capture the spatial and temporal variability of temperature in the region is through the use of satellite data [52]. NOAA has since adapted the combined use of in situ and satellite AVHRR data through its National Centers for Environmental Information (NCEI) facility. Daily optimum interpolated SSTs are provided regularly and can be downloaded at https://www.ncei.noaa.gov/products/optimum-interpolation-sst. These data have been validated and compared with other data sources such as those from Aqua-Moderate Resolution Imaging Spectroradiometer (MODIS) [53]. Monthly SST data were only utilized where sea ice concentrations are <10% and reported as missing data otherwise.

### Surface nitrate data

In order to provide ancillary seasonal context for the DBO sites, spatial data of monthly surface nitrate concentrations were obtained from the World Ocean Atlas [54]. Mean surface nitrate concentration extracted for each DBO site for each month was calculated using the one-degree spatial resolution data. The historical oceanographic data used in this dataset were obtained from the NCEI/World Data Service for Oceanography archives and include all data gathered as a result of the Global Oceanographic Data Archaeology and Rescue and World Ocean Database projects. While detailed nutrient data have been collected via DBO cruise efforts over the past decade (primarily during summer months), we utilize the World Ocean Atlas data here not to provide a detailed assessment of nutrient availability across the DBO but rather to provide a broad, simplified perspective of the typical seasonality of nutrient concentrations across the region. A comparison of recent summer in situ surface nitrate data showed good consistency with World Ocean Atlas data across DBO sites.

### Sea ice concentration, timing of breakup/formation, and annual persistence

Daily sea ice concentration data were obtained from the Scanning Multi-channel Microwave Radiometer (SMMR), Special Sensor Microwave/Imager (SSM/I), and Special Sensor Microwave Imager/Sounder (SSMIS) passive microwave instruments, calculated using the Goddard Bootstrap (SB2) algorithm [55,56]. As SMMR data are only available every other day, they were first linearly interpolated to create a full daily time series. As is standard in many prior studies, we used a 15% sea ice concentration threshold to define the presence vs. absence of sea ice cover for calculations of sea ice extent. To calculate annual sea ice persistence, we then summed the number of days when sea ice was present (for each pixel) over each September 15 through September 14 (of the following year) time period in order to capture a full sea ice season (which would not be the case if a calendar year metric was utilized instead). The timing of sea ice breakup (or formation) was determined by identifying the date on which a pixel registered two consecutive days below (or above) a 15% sea ice concentration threshold, where we defined the breakup period as March 15–September 14 and the formation period as September 15–March 14. Requiring two consecutive days of the breakup/formation condition ensured that the defined sea ice events were persistent rather than spurious occurrences.

### Satellite chlorophyll-a concentrations, primary productivity, and photosynthetically available radiation

There have been several sources of chlorophyll-*a* concentration data that have been available in recent years, but for consistency and long-term coverage we use data from Aqua-MODIS (launched in 2002 and in operation up to the present). The sensor has 36 channels covering the electromagnetic spectrum from ultraviolet to thermal-infrared wavelengths at resolutions from 250 to 1000 m. Chlorophyll-*a* concentrations used in this study (Aqua-MODIS Reprocessing 2018.0 chlor_a algorithm) were derived from MODIS calibrated radiances using two algorithms as described by NASA: the OC3m algorithm that was developed at NASA Goddard Space Flight Center (GSFC) and makes use of band ratios and in situ measurements as described by O’Reilly et al. [57] and the CI algorithm that makes use of reflectance differences in conjunction with a model as described by Hu et al. [58]. The data are made available by the Ocean Biology Processing Group and were downloaded from the GSFC Distributed Active Archive Center (DAAC) at https://oceandata.sci.gsfc.nasa.gov/directdataaccess/Level-3%20Mapped/Aqua-MODIS. Daily chlorophyll-*a* image data were averaged for each month to create a monthly chlorophyll-*a* dataset. Nearest neighbor averaging was used to fill in interstitial values (a minimum of seven pixels surrounding a missing pixel was required in order for filling to occur). The monthly chlorophyll-*a* concentration image data were then combined with SST data, as described below, and ancillary datasets to derive monthly net primary productivity using the technique described by Behrenfeld and Falkowski [59]. The resulting primary productivity data developed in this study are henceforth referred to as the Goddard Space Flight Center primary productivity (GSFC PP) dataset. Monthly chlorophyll-*a* and primary productivity data were only utilized where sea ice concentrations were <10% and were reported as missing data otherwise. Annual primary productivity (g C/m^2^/year) was further calculated using monthly data from March through September only. The satellite-based primary productivity data presented in this study only represent open ocean values and do not represent other sources of productivity in Arctic Ocean waters that include sea ice algal production [22,23], under-ice phytoplankton [19,20], or deep chlorophyll maxima [18]. However, recent studies have begun to use satellite remote sensing to estimate sea ice primary productivity, e.g., [60]. Further challenges include the inability of satellite sensors to collect ocean color satellite data at high latitudes owing to the presence of sea ice and/or cloud cover. While monthly data may have similar problems for each year, they still provide a viable first-order estimate of interannual change over time.

Spatial variability in the signature of chlorophyll-*a* has been reported in the literature. This is especially the case when global data, derived using algorithms that made use of in situ data mainly from tropical and adjacent regions, were compared with data collected in the Arctic and Antarctic regions. Normalization parameters have been derived to make satellite data consistent with in situ data collected in the western Arctic [61,62]. However, when the same normalization parameters were applied to satellite data in the eastern Arctic, the results showed large overestimates when compared with in situ data from the region. Using a regional algorithm also creates the problem of locating boundaries between global data and regional data. For lack of a better alternative, the data used in this study make use of the global dataset for consistency and to avoid the aforementioned problems. Similar methods have also been used in the Antarctic for analogous studies [63]. In particular, preliminary comparisons between the Aqua-MODIS chlorophyll-*a* concentrations and in situ observations at our DBO sites show correlation coefficients of up to ~0.7 (similar to other comparisons across the Arctic, e.g., [64]) where satellite observations slightly underestimate in situ measurements, particularly at higher chlorophyll-*a* concentrations (>1 mg/m^3^). We additionally compared the same in situ chlorophyll-*a* measurements with the GlobColour chlorophyll-*a* satellite product (https://globcolour.info/) to assess whether a merged product would provide improved results. Although correlation coefficients and standard deviations were nearly identical between the two (i.e., Aqua-MODIS vs. in situ and GlobColour vs. in situ), slopes and intercepts were better for Aqua-MODIS (1.30 and 0.12, respectively) than for GlobColour (1.45 and 0.16, respectively). As such, we chose to utilize the Aqua-MODIS chlorophyll-*a* data rather than the merged GlobColour product across the DBO sites. Furthermore, trends over time of in situ chlorophyll-*a* concentrations at DBO sites 1–5 [65] show similar patterns to our trends in satellite observations, further justifying the use of the global Aqua-MODIS chlorophyll-*a* product in this study.

**Fig 2:**
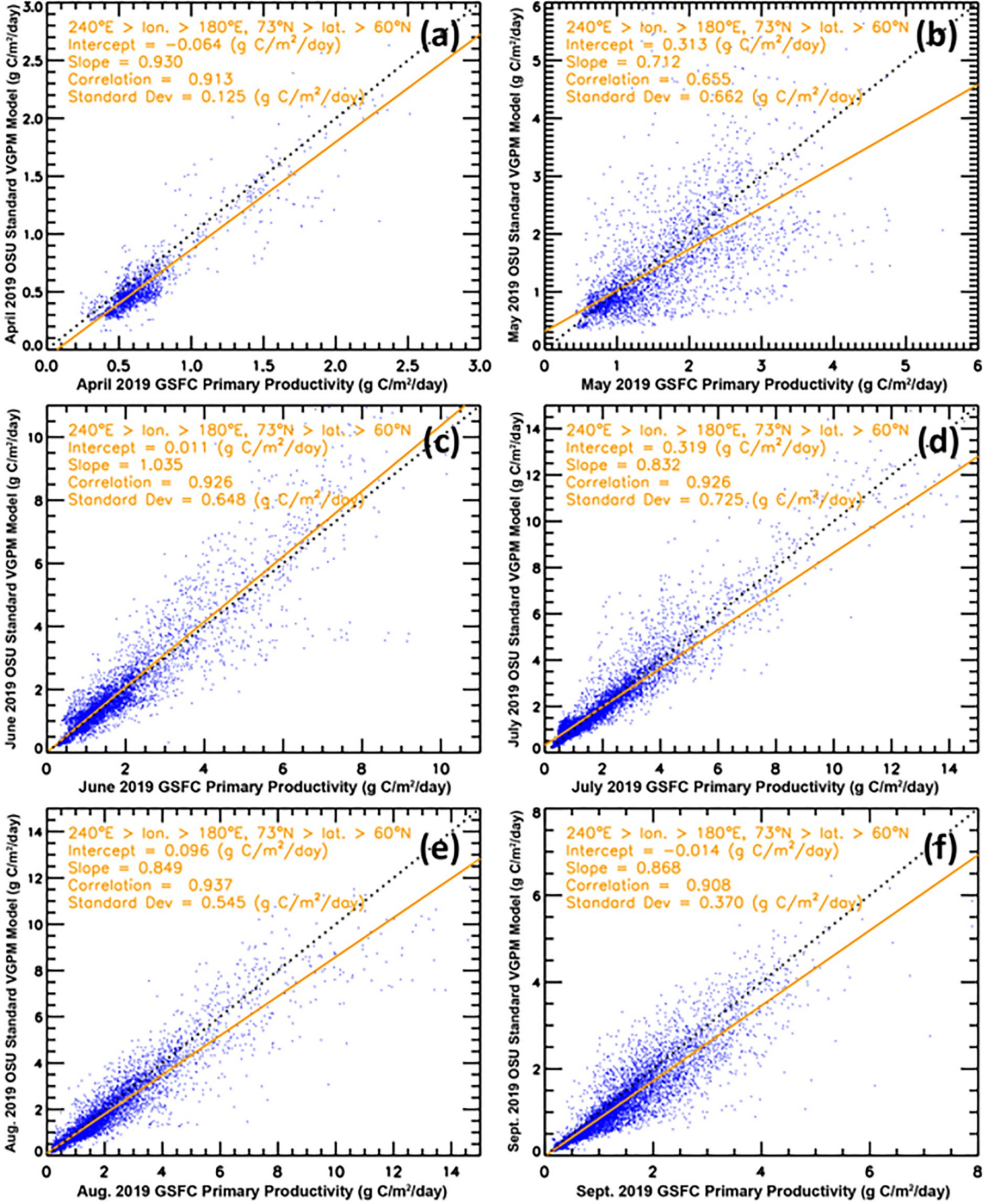
Scatterplot comparisons of the Oregon State University (OSU) primary productivity data compared to the primary productivity values in this study over the Pacific Arctic study region for (a) April, (b) May, (c) June, (d) July, (e) August, and (f) September of 2019.

**Fig 3:**
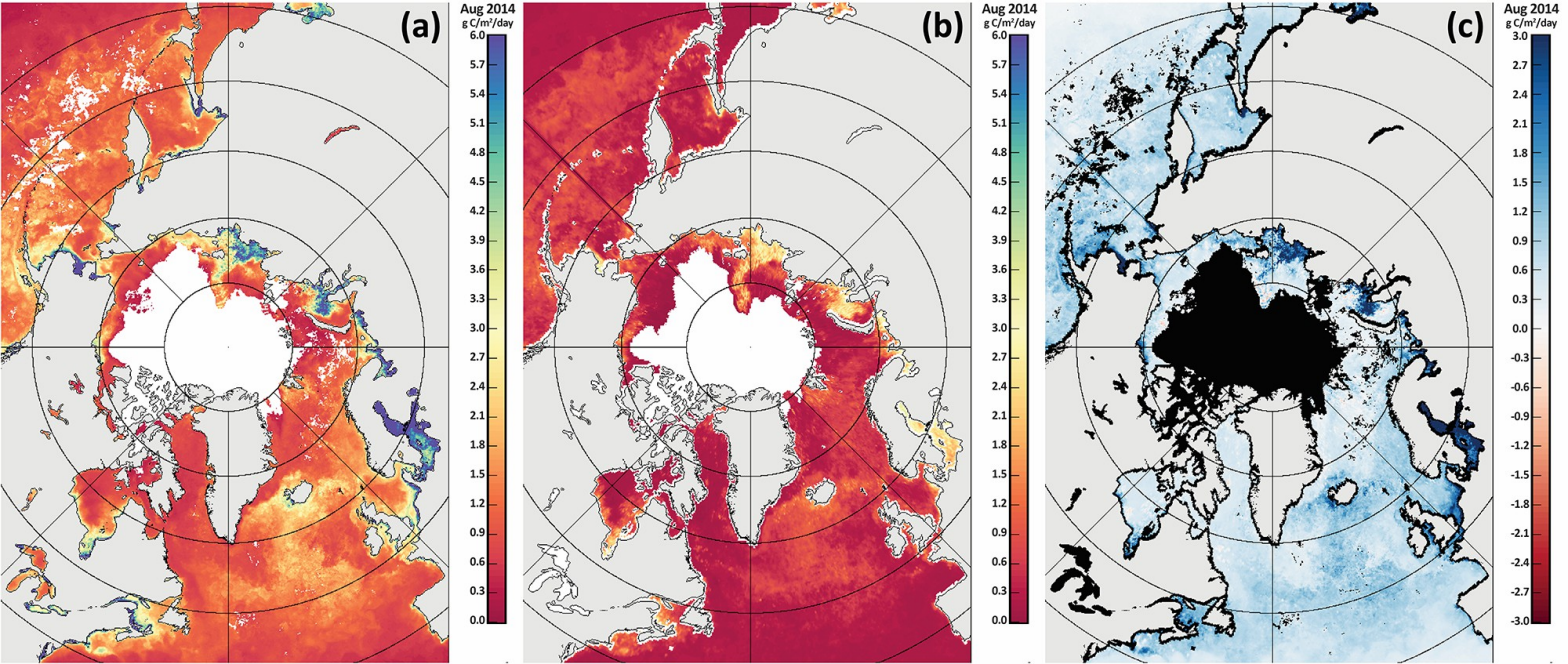
Maps of the pan-Arctic region for August 2014 showing (a) primary productivity values as derived in this study, (b) primary productivity values as derived by Kahru et al. (2016), and (c) the difference map of (a)–(b). Basemap datasets from Natural Earth [49] and ESRI [50].

**Fig 4:**
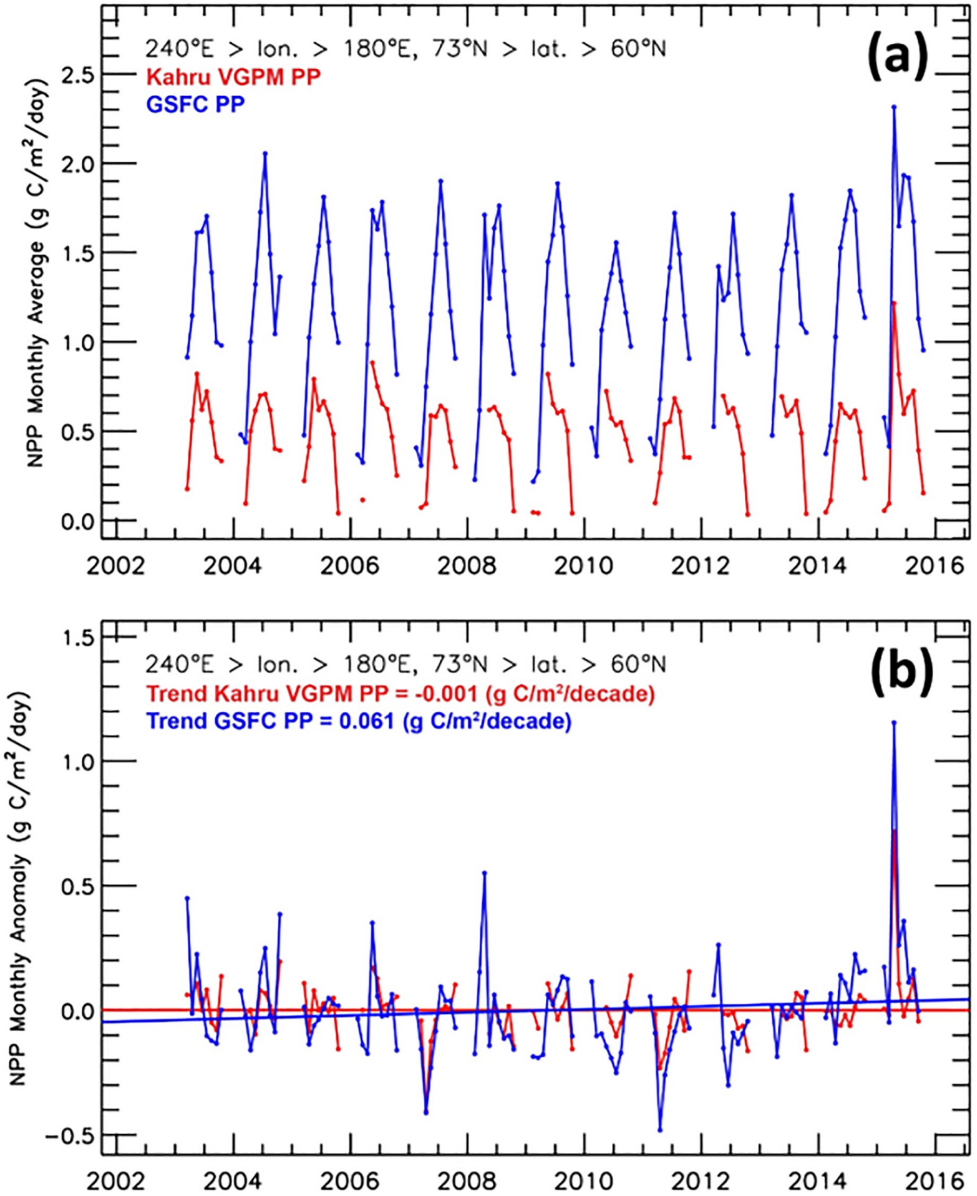
Time series of primary productivity derived in this study (GSFC PP) and those derived from Kahru et al. (2016) [45] (Kahru VGPM PP) across the Pacific Arctic study region for (a) monthly average values of primary productivity and (b) monthly anomalies of primary productivity.

There are three widely used satellite-based primary productivity models that include the vertically generalized production model (VGPM), the carbon-based productivity model (CBPM), and the carbon, absorption, and fluorescence euphotic-resolving (CAFE) model, e.g., [64]. In particular, the VGPM is a well-known model that ranks highly in terms of model-to-model comparisons [45,48,64] and additionally includes publicly available primary productivity time series [45,66] against which we can compare our primary productivity image time series. To this end, we compared our GSFC PP data with the Oregon State University (OSU) VGPM data [66] as well as the Kahru et al. (2016) VGPM data [45] (Figs 2, 3 and 4) for the Arctic and Pacific Arctic regions. In comparisons with the OSU VGPM dataset (Fig 2), it is apparent that there is good agreement of the two datasets with correlation coefficients ranging from 0.903 to 0.937, except for the month of May. Discrepancies may be due to the OSU data utilizing MODIS SST data and our data utilizing the Optimum Interpolation SST dataset. Because of this, we did not find any reason to utilize the OSU data, particularly since our GSFC PP product makes use of improved sea ice concentration data (i.e., the Bootstrap (SB2) algorithm) to mask out data where sea ice concentrations were >10% and SST (i.e., the Optimum Interpolation SST dataset) that has been validated to agree with in situ measurements in the Arctic [67]. An additional key issue is the presence of chromophoric dissolved organic matter (CDOM) across the Pacific Arctic region [68], which (among others) is taken into consideration by a recent study investigating the impact of sea ice on primary productivity across the Arctic Ocean [45] (henceforth referred to as the Kahru PP dataset). A comparison of color-coded maps of primary productivity using the Kahru PP dataset and our GSFC PP dataset (Fig 3) shows that the Kahru PP values are consistently lower than ours. Our concern with the Kahru PP data is that even in deep ocean (Case 1) waters (where CDOM is expected to be negligible), the Kahru PP data are still lower (Fig 3c). This suggests that the Kahru PP data may be overestimating CDOM (and underestimating primary productivity) in many locations across the Arctic region. The satellite-based chlorophyll-*a* data used in our study has been well-validated in Case 1 waters and for this reason, we use our estimates of primary productivity (GSFC PP) in this study. Larger areas of discrepancy occur in the vicinity of river mouths such as the Yukon and Mackenzie (Fig 3c), however the datasets are more similar to one another within our defined DBO sites. For completeness, we present a comparison of time series data to show quantitative differences of our GSFC PP data and the Kahru PP data across the Pacific Arctic study region (Fig 4). While interannual variability and trends are similar between these two datasets, it is likely that actual primary productivity values lie somewhere between these two datasets.

While PAR is incorporated as an ancillary dataset into the GSFC PP algorithm here, a direct assessment of shifting measurements of PAR was also undertaken to give insight into changing atmospheric conditions (e.g., [69,70]) across the DBO sites and Pacific Arctic sector. We acquired monthly (March through September) PAR data over the years 2003–2020 from the GSFC DAAC at https://oceandata.sci.gsfc.nasa.gov/directdataaccess/Level-3%20Mapped/Aqua-MODIS. Monthly PAR data were subsequently averaged into climatological means with spatial trends (using the Theil-Sen median slope estimator) calculated over the 2003–2020 period, as described in greater detail below.

### Statistical analyses

Our statistical analyses were applied to all time series datasets consistently. First, monthly mean values of SST, sea ice concentration, chlorophyll-*a* concentration, primary productivity, and PAR (as described above) were compiled over the years 2003–2020, which is the time period when Aqua-MODIS data are available (i.e., the first full year of data began in 2003). Annual mean values of primary productivity (March–September only), the timing of sea ice breakup and formation, and sea ice persistence were additionally calculated across the region for the years 2003–2020. Next, spatial trends using the Theil-Sen median slope estimator [71] were calculated for SST, sea ice concentration, sea ice breakup/formation/persistence, chlorophyll-*a*, primary productivity, and PAR data. The Theil-Sen trend uses a robust non-parametric trend operator that is particularly well suited for assessing the rate of change in noisy and/or short time series [72], which in this study is 18 years. The statistical significance of the Theil-Sen trends (*p*<0.1) was established using the non-parametric Mann-Kendall test for monotonic trend [73,74]. For those datasets that include missing data (SST, chlorophyll-a, primary productivity, PAR, and sea ice breakup/formation i.e., when no ice or multi-year ice was present for a given year), we show only those trends for pixels that had at least 71% of the time series present (or in the case of this study, 13 of the 18 time steps). This requirement ensures that only robust trends are reported, given that the “breakdown bound” for the Theil-Sen trend is 29% (meaning that unknown or potentially “wild” values would have to persist for more than 29% of a time series in order to affect the overall trend values [72]). Lastly, we calculated the number of open water days per year (March–September only; defined as days with sea ice concentrations <15%) in order to perform a linear regression with annual primary productivity across the region. Spatial representations of the slope as well as coefficient of determination of the annual primary productivity vs. open water days relationship were calculated to establish the strength of this linear regression.

## Results

### Climatological means of environmental variables

**Fig 5:**
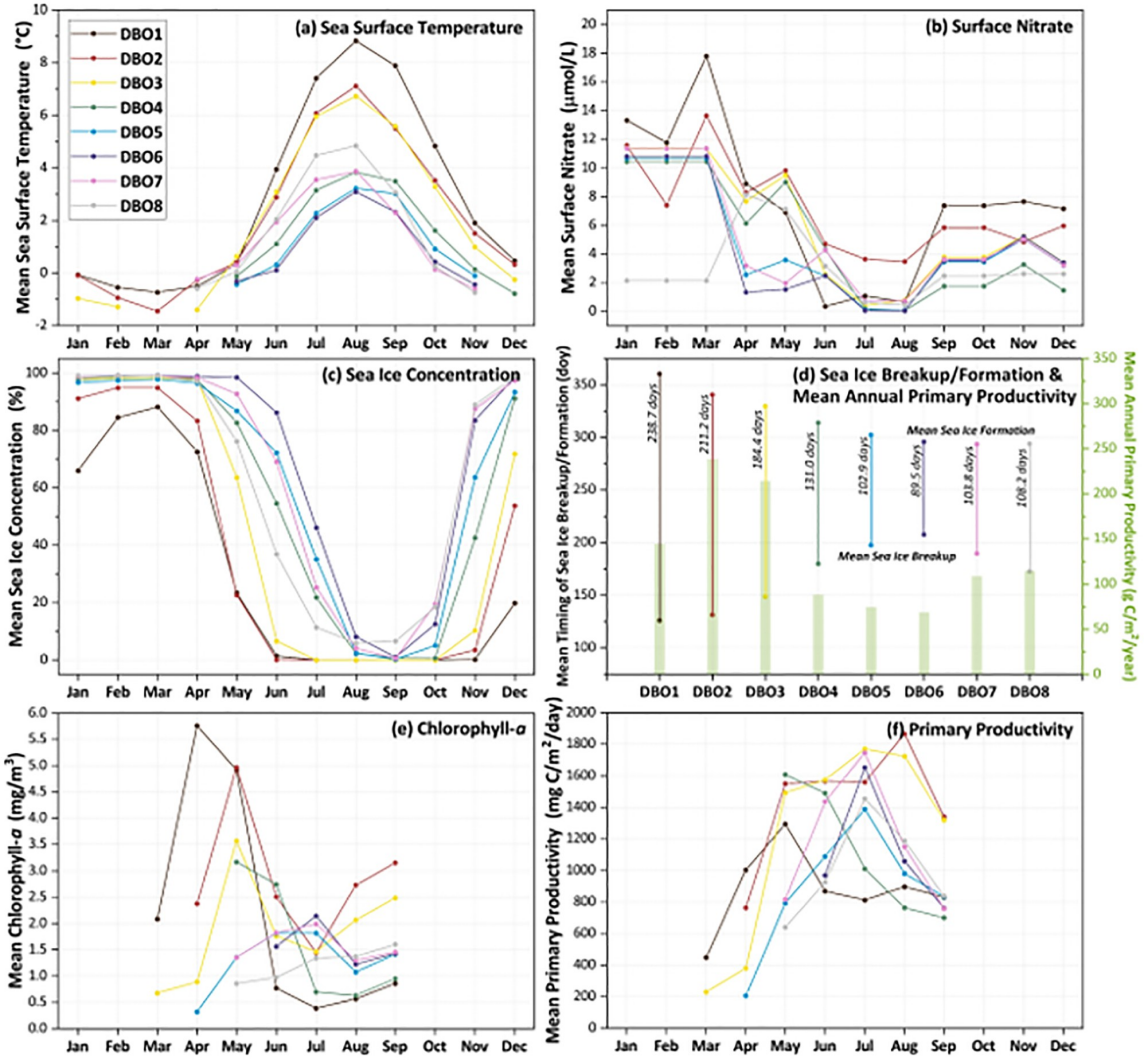
Mean values for each of the eight DBO sites (over the years 2003–2020) for (a) satellite-derived monthly sea surface temperatures, (b) world ocean atlas-derived monthly surface nitrate concentrations, (c) satellite-derived monthly sea ice concentrations, (d) satellite-derived timing of sea ice breakup and formation (with the mean number of annual open water days also designated), (e) satellite-derived monthly chlorophyll-*a* concentrations, and (f) satellite-derived monthly primary productivity. Color coding for DBO sites designated in (a) is the same for all composite plots.

The climatological mean values (2003–2020) of environmental variables investigated (SST, surface nitrate, sea ice concentration, timing of sea ice breakup/formation, chlorophyll-*a* concentration, and primary productivity) show a distinct latitudinal gradient across all DBO sites as well as strong seasonality within each individual DBO site (Fig 5). SST values in general are warmest for DBO1 (with a mean value of ~9°C in August), coolest for DBO6 (with a mean value of ~5°C in August), and trend slightly warmer again east of DBO6 for both DBO7 and DBO8 (Fig 5a and S1 Fig). Surface nitrate concentrations show clear seasonal patterns as well, with relatively high values during January through March, declining from April through August as biological productivity utilizes nutrients, then increasing slightly again during September through December (Fig 5b) as upwelling and/or wind mixing brings nutrients back to the surface despite cooling temperatures and diminished sunlight availability. Sea ice concentrations show similar but antisymmetric patterns to SSTs, with the longest seasonally ice-free period at DBO1, and trending shorter to the north with the shortest ice-free period at DBO6, and then shifting to longer ice-free periods again at DBO7 and DBO8 (Fig 5c and S2 Fig). Sea ice was additionally analyzed into annual values that reflect timing of breakup/formation (and therefore mean number of open water days per year), which can be compared with mean annual values of primary productivity (Fig 5d and S3 Fig). Mean annual sea ice persistence varies from only a few days at the southern ice edge in the Bering Sea to 365 days/year north in the Canada Basin northwest of the Canadian Archipelago (S3a Fig). The mean timing of sea ice breakup occurs continually later, moving northward from DBO1-DBO6, then becomes slightly earlier again for DBO7 and DBO8 in the vicinity of the Mackenzie River delta and Cape Bathurst Polynya (S3b Fig). The mean timing of sea ice formation occurs over a generally shorter timeframe than breakup across the region and occurs later in the year moving southward as cooler temperatures progress seasonally (S3c Fig). Given the timing of sea ice breakup/formation, the mean length of the open water season over the 2003–2020 period is 238.7 days (DBO1), 211.2 days (DBO2), 184.4 days (DBO3), 131.0 days (DBO4), 102.9 days (DBO5), and 89.5 days (DBO6), with the length increasing again eastward from there following the pattern of ice retreat with 103.8 days (DBO7) and 108.2 days (DBO8) (Fig 5d). Notably, DBO1 is located in the St. Lawrence Island Polynya region (prominent during winter) and DBO8 is located in the Cape Bathurst Polynya region (prominent during spring).

Monthly mean values of chlorophyll-*a* (Fig 5e and S4 Fig), primary productivity (Fig 5f and S5 Fig), and PAR (S6 Fig) give insight into the seasonal variability of these parameters as well. DBO1, DBO2, and DBO3 exhibit similar patterns in chlorophyll-*a* concentrations, with the highest concentrations in early spring (April-May), falling in June-July, then increasing again in August and September (although these late season chlorophyll-*a* concentrations are close to double for DBO2 and DBO3 than at other sites). Chlorophyll-*a* concentrations at sites DBO4-DBO8 show seasonal variability, however over a shorter season given the available ice-free data at these sites. In particular, DBO4 exhibits its highest concentrations during May-June, lowest concentrations in June-July, then a slight uptick in September. DBO5 and DBO7 show similar chlorophyll-*a* patterns with increases from May-July, falling in August, then increasing slightly in September. DBO6 exhibits only four months of sea ice-free chlorophyll-*a* data, increasing from June-July, decreasing in August, then increasing slightly in September. DBO8 is the only site that does not exhibit a mid-summer decrease in chlorophyll-*a* concentrations, continuously increasing over the ice-free months at that location, from May-September. Corresponding rates of primary production (Fig 5f and S5 Fig) show strong seasonal patterns as well, although a secondary autumn peak in primary productivity (as seen with chlorophyll-*a* concentrations) is much less apparent for the DBO sites in general. This is likely at least in part due to cooling SST in September and October not contributing to as high primary productivity compared to earlier in the season (June-August). Similarly, while chlorophyll-*a* concentrations tend to peak early in the season (within weeks of sea ice breakup), primary productivity tends to peak later in the season (despite lower chlorophyll-*a* concentrations) perhaps because of the contributions of warmer mid-summer SST to these rates and/or varying photosynthetic rates among phytoplankton groups [15]. Annual primary productivity additionally gives insight into the biological variability among DBO sites, with the highest values at DBO2 (238.7 g C/m^2^/year) and DBO3 (214.5 g C/m^2^/year); the lowest values at DBO4 (88.3 g C/m^2^/year), DBO5 (74.5 g C/m^2^/year), and DBO6 (68.7 g C/m^2^/year); and middle-range values at DBO1 (144.5 g C/m^2^/year), DBO7 (108.9 g C/m^2^/year), and DBO8 (114.6 g C/m^2^/year) (Fig 5d). Clear seasonal variability in PAR is apparent across the region as well, with the highest values during June and the lowest values during March and September (over the investigated March through September period) (S6 Fig).

### Trends in environmental variables

**Fig 6:**
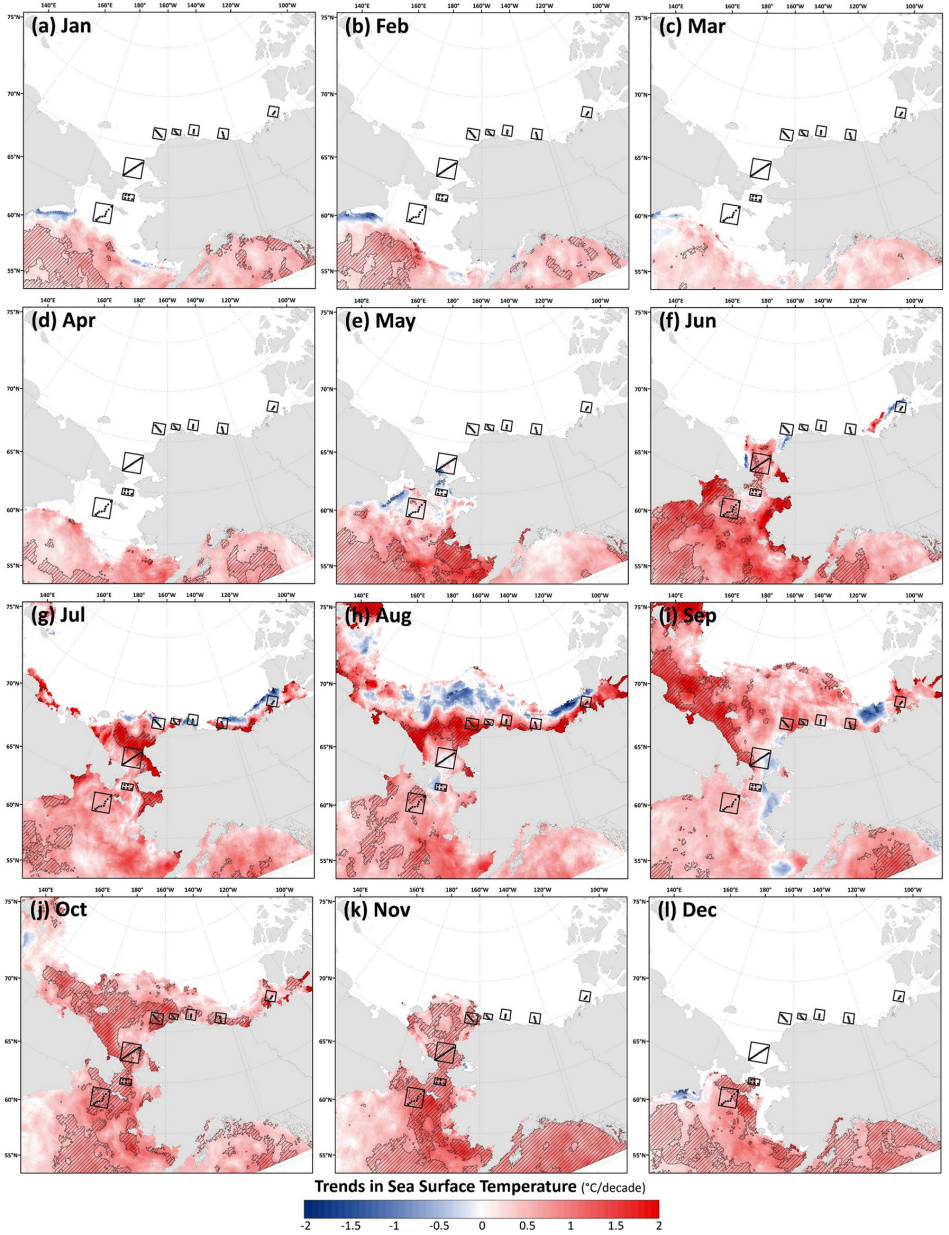
Theil-Sen median trends in satellite-derived monthly sea surface temperature (SST) where the hatched pattern indicates a statistically significant Mann-Kendall test for trend (*p*<0.1) over the 2003–2020 period. Basemap datasets from Natural Earth [49] and ESRI [50].

After establishing the mean seasonal behavior of environmental variables across the Pacific Arctic region and the DBO sites, it is also useful to quantify rates of change in SST, sea ice cover, chlorophyll-*a*, primary productivity, and PAR. As such, the Theil-Sen formula is used to estimate the median trends for all variables for each month, and annually in the case of sea ice persistence/breakup/formation and annual primary productivity. For SST, warming trends are pervasive across the region for all months (Fig 6), with the steepest trends during June in the Bering Sea, July and August in the Chukchi Sea, and September in the East Siberian Sea. Such trends in summer temperature contributed in part to the rapid decline in multiyear ice, as reported earlier. Widespread and statistically significant warming trends are additionally present across the region during October and November in the Bering and Chukchi Seas, and during December in the Bering Sea. These trends in autumn (particularly in October) caused the onset of freeze-up to occur at later dates in more recent years. We additionally find isolated areas of cooling (although not statistically significant) along the northern sea ice edge in July and August in the Chukchi and Beaufort Seas, in Alaskan coastal waters and north of the Mackenzie River mouth in September, and along the Siberian coast south of the Gulf of Anadyr in December, January, and February.

**Fig 7:**
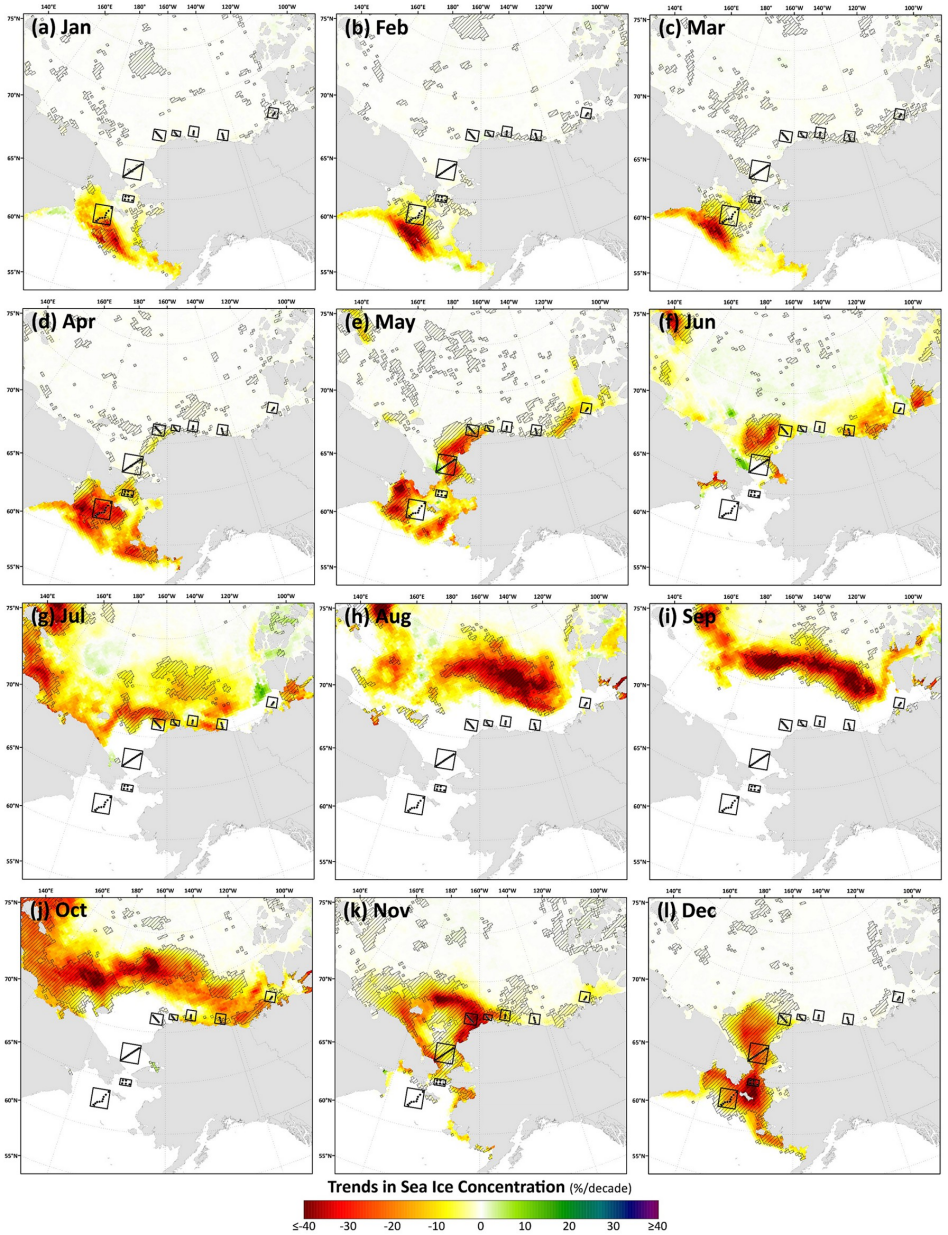
Theil-Sen median trends in satellite-derived monthly sea ice concentration where the hatched pattern indicates a statistically significant Mann-Kendall test for trend (*p*<0.1) over the 2003–2020 period. Basemap datasets from Natural Earth [49] and ESRI [50].

**Fig 8:**
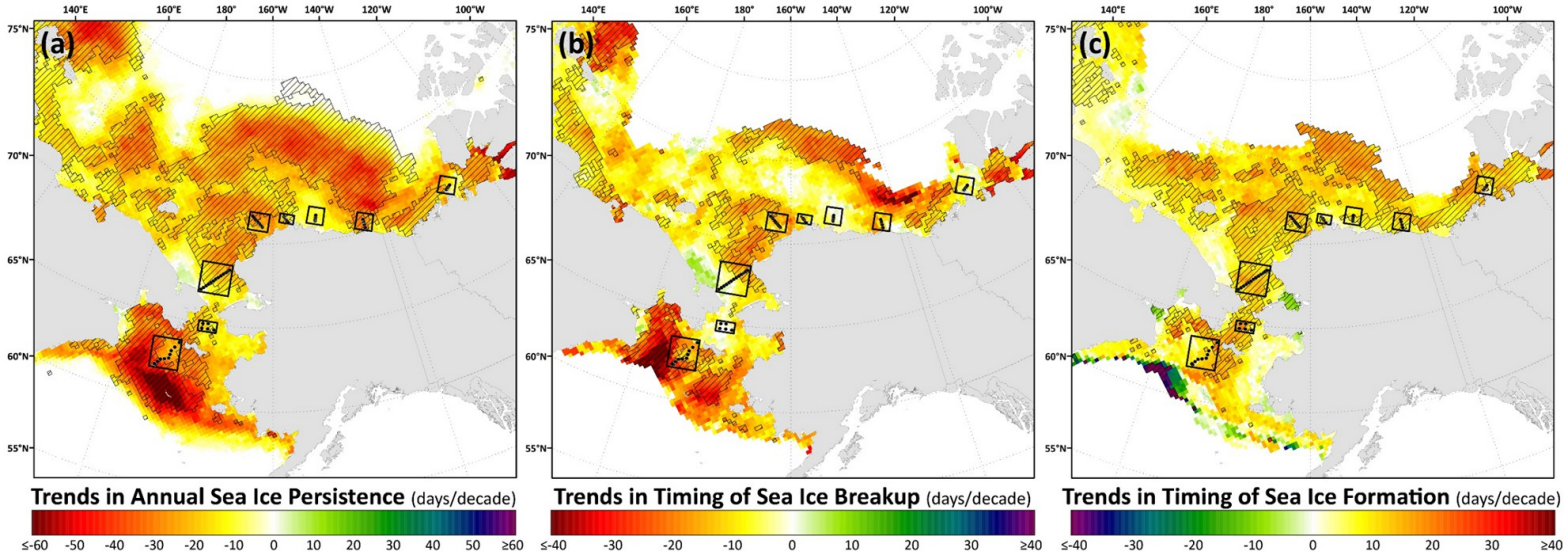
Theil-Sen median trends in sea ice events across the Pacific Arctic region: (a) annual sea ice persistence, (b) timing of sea ice breakup, and (c) timing of sea ice formation over the 2003–2020 period. The hatched pattern indicates a statistically significant (*p*<0.1 using the Mann-Kendall test for trend) over the 2003–2020 period. Basemap datasets from Natural Earth [49] and ESRI [50].

Trends in sea ice concentrations show decreases in all months (Fig 7), congregating around the sea ice edge in the Bering Sea during January-March, moving northward from the Bering to the Chukchi from April-July, and moving even farther north (well north of the DBO sites) into the Canada Basin during August-October when the DBO sites are all still typically open water. Significant decreases in sea ice concentrations again develop at the DBO sites in November and December, as the timing of sea ice formation has been delayed in these locations. These phenological patterns can be more easily seen in trends of annual sea ice persistence and the timing of sea ice breakup/formation (Fig 8), where the steepest trends observed are those for reductions in annual sea ice persistence and earlier timing of breakup in the Bering Sea in the vicinity of DBO1. Trends in the timing of breakup tend to generally show more heterogeneity than the more consistent ~10 days/decade later trends for sea ice formation that are observed at most DBO sites. The spatial trends observed in the timing of sea ice breakup/formation and annual persistence of sea ice cover can additionally serve as a guide for identifying locations where the duration of open water has been increasing significantly (Fig 8).

While observations of the monotonic trends in sea ice cover can be insightful, the interannual variability in ice cover can be very different from the longer term (e.g., decadal) variability (e.g., Fig 1). During winter (March), there is little variability in the average locations of the ice edge over decadal periods (Fig 1). In summer (September), the variability is more significant and reflects the monotonic retreat of the summer ice cover from the earliest to the more recent decades. On the other hand, the ice edge contours for the years 2018, 2019 and 2020 show that the ice edge was much more variable in recent years, with the winter 2018 ice edge farthest to the north, followed by 2019. The reverse is true during the summer period in the central Arctic with the year 2020 furthest north (except west of the longitude 120°W), followed by 2019 and 2018. This suggests that the yearly influence of sea ice cover on primary productivity can be very different on a year-to-year basis compared to that owing to long term changes.

**Fig 9:**
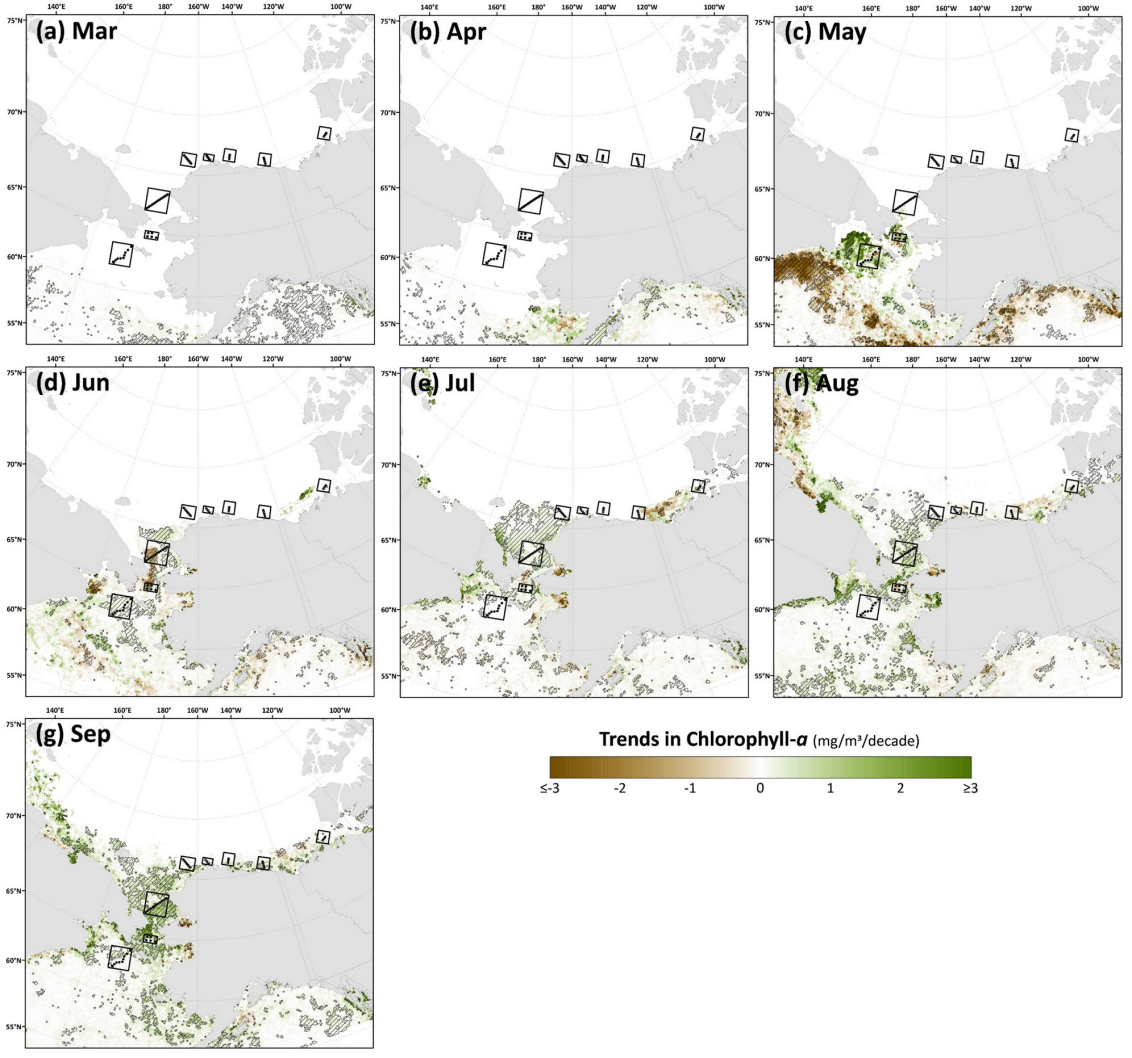
Theil-Sen median trends in satellite-derived monthly chlorophyll-*a* concentrations where the hatched pattern indicates a statistically significant (*p*<0.1 using the Mann-Kendall test for trend) over the 2003–2020 period. Basemap datasets from Natural Earth [49] and ESRI [50].

**Fig 10:**
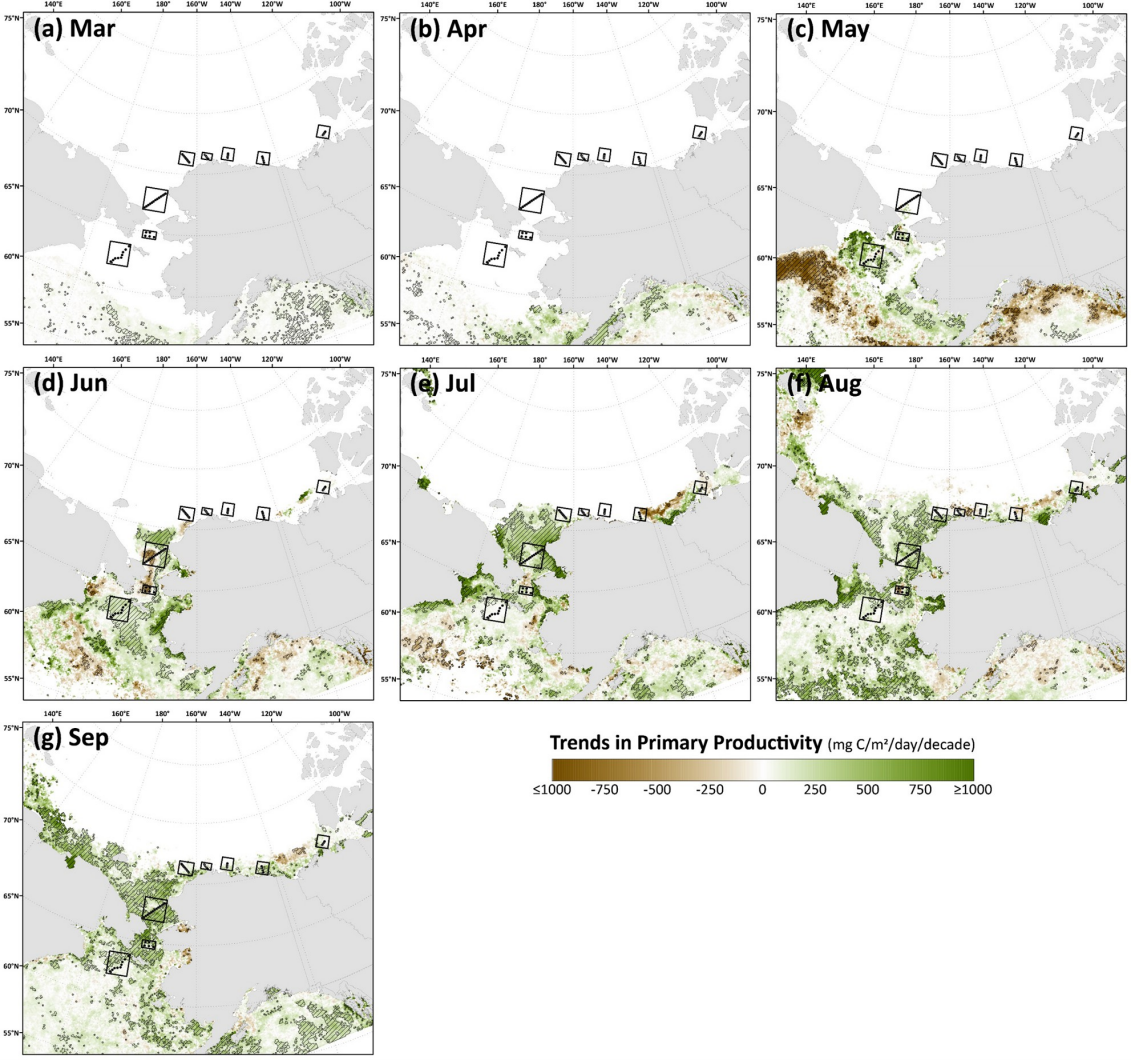
Thiel-Sen median trends in satellite-derived monthly primary productivity where the hatched pattern indicates a statistically significant (*p*<0.1 using the Mann-Kendall test for trend) over the 2003–2020 period. Basemap datasets from Natural Earth [49] and ESRI [50].

**Fig 11:**
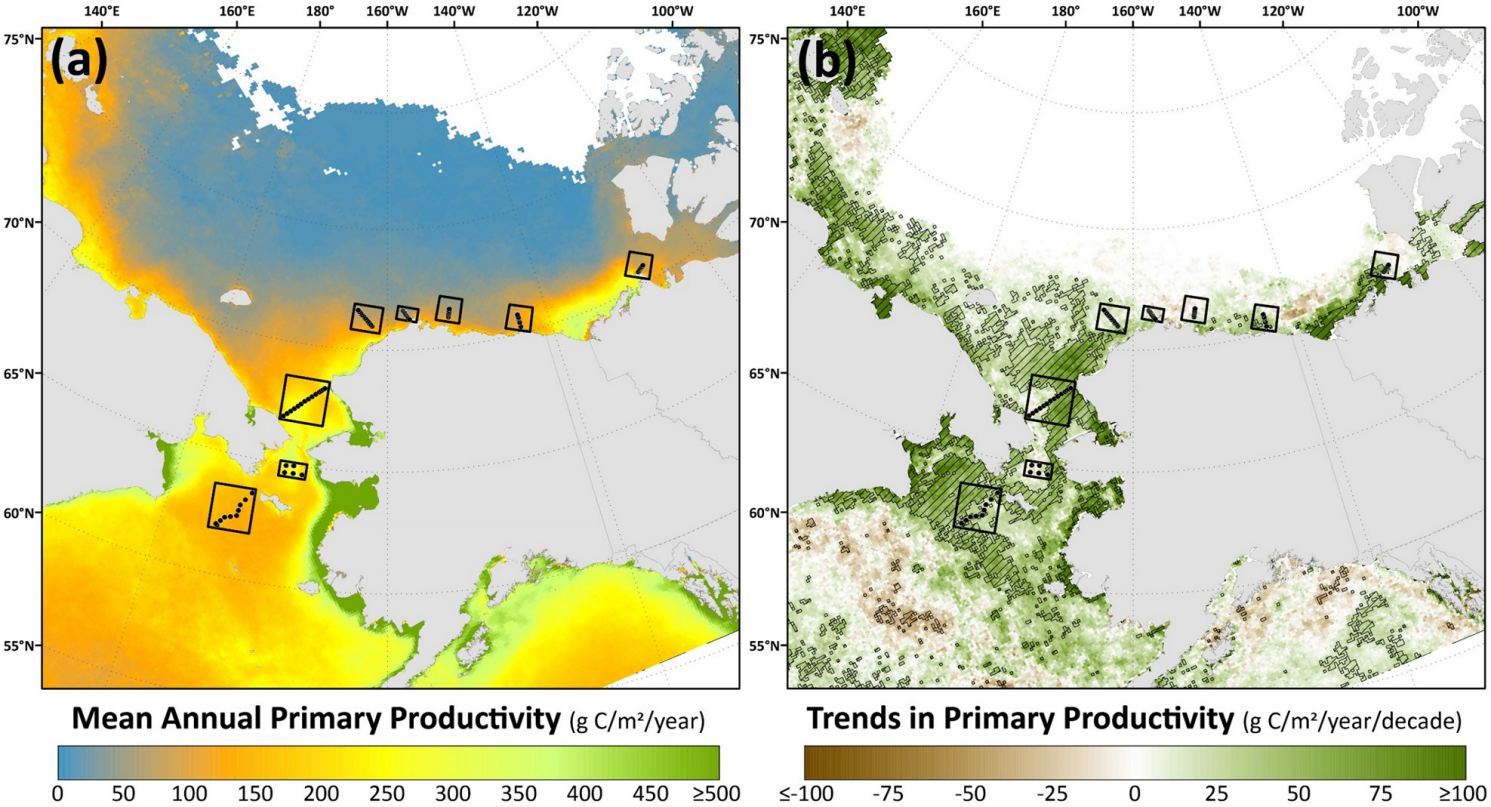
(a) Mean annual (March-September) primary productivity and (b) Theil-Sen median trends in annual (March-September) primary productivity where the hatched pattern indicates statistically significant trends (*p*<0.1 using the Mann-Kendall test for trend) over the 2003–2020 period. Basemap datasets from Natural Earth [49] and ESRI [50].

**Fig 12:**
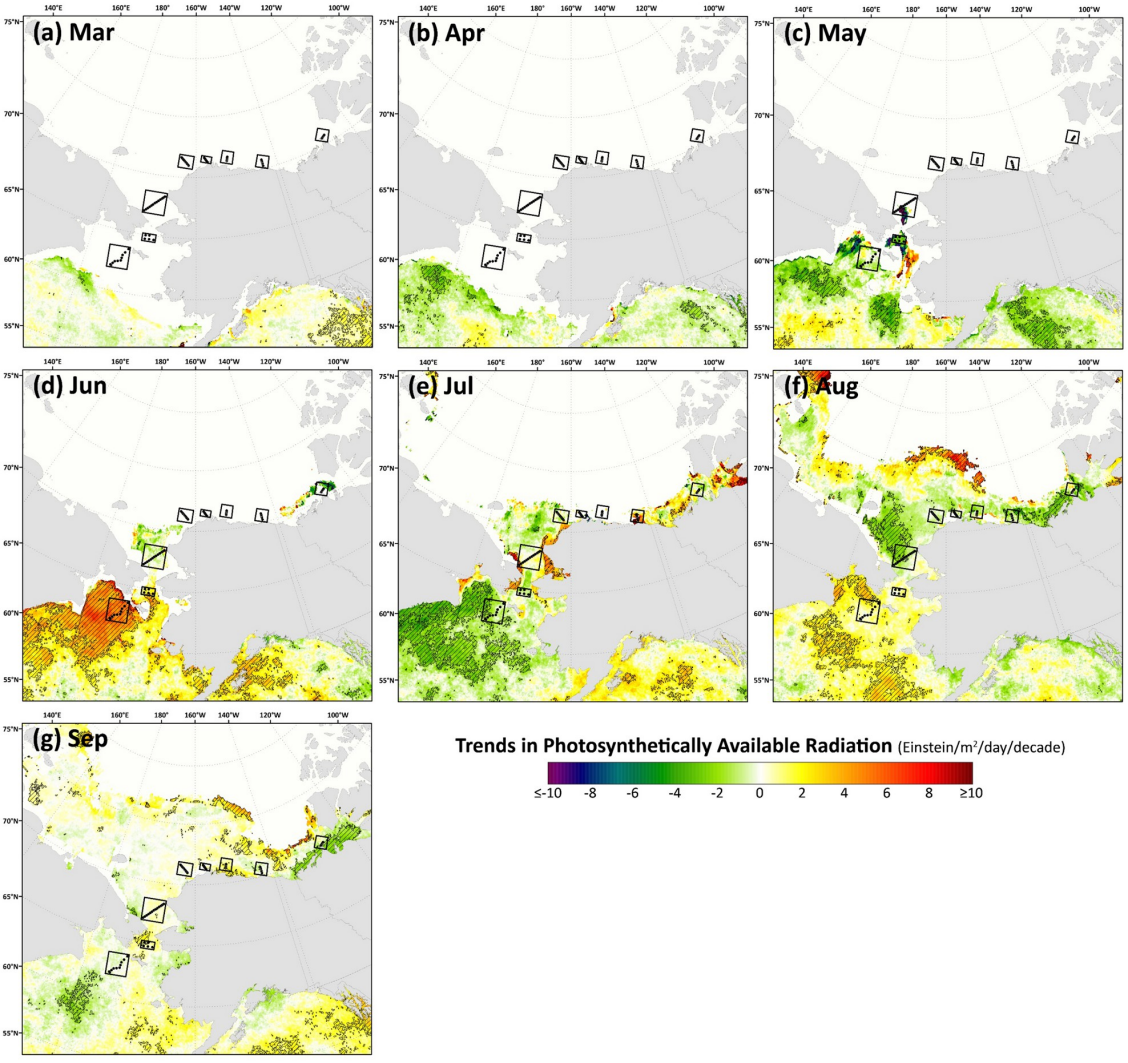
Thiel-Sen median trends in satellite-derived monthly photosynthetically available radiation where the hatched pattern indicates a statistically significant (*p*<0.1 using the Mann-Kendall test for trend) over the 2003–2020 period. Basemap datasets from Natural Earth [49] and ESRI [50].

Spatial monthly Theil-Sen trends in chlorophyll-*a* (Fig 9) and primary productivity (Fig 10) show similar patterns to one another across the Pacific Arctic region, although trends in primary productivity are generally more significant and geographically widespread as these changes are not only incorporating rising chlorophyll-*a* concentrations but are amplified by warming SST as well. During May, an interesting juxtaposition is observed with negative trends in chlorophyll-*a*/primary productivity along the Siberian coast south of the Gulf of Anadyr and positive trends in both parameters just to the northeast, near DBO1, where some of the steepest trends are present throughout the year. In June, significant decreases in chlorophyll-*a*/primary productivity emerge in DBO2 and the western portion of DBO3 in the Bering Strait region. Otherwise, most other statistically significant trends for productivity and chlorophyll-*a* are positive and located in the northern Bering Sea (north of St. Lawrence Island) and the southern Chukchi Sea during July, August, and September. Fewer statistically significant trends occur during October, but data are spatially limited during this month owing to the onset of polar night. Compiling data into trends of annual primary productivity also helps to identify regions of greatest importance of change (Fig 11). Annual primary productivity exhibits most of its significant trends in the northern Bering and southern Chukchi Seas, although the steep negative trends in chlorophyll-*a*/primary productivity for DBO2 and the western half of DBO3 during June seem to counterbalance any overall annual increases in those areas that would be present otherwise (Figs 9b and 10b). The DBO4-DBO7 sites are located in areas that exhibit less significant change in annual primary productivity, although DBO8 is adjacent to an important region of productivity change in the eastern Beaufort Sea. Furthermore, positive trends in PAR are most apparent for DBO1 during June (Fig 12d), whereas negative trends in PAR are most apparent for the western sector of DBO1 during July (Fig 12e) and the western sector of DBO3 during August (Fig 12f).

**Fig 13:**
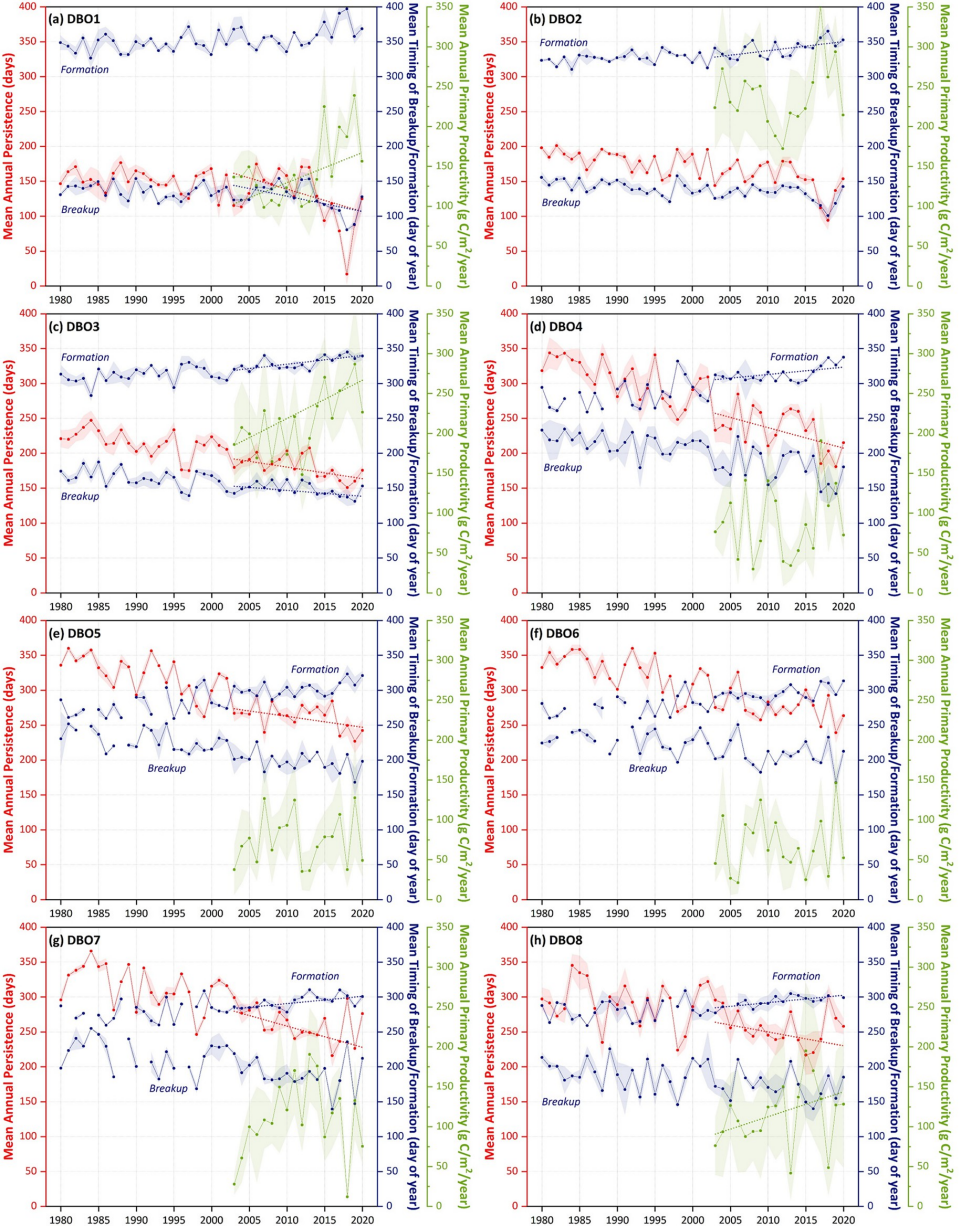
Annual sea ice persistence, timing of sea ice breakup/formation, and annual primary productivity for all eight DBO sites. Trend lines (Sen median slope) are only shown for those time series (over the years 2003–2020) that show statistically significant trends (*p*<0.1 using the Mann-Kendall test for trend). Error bar shading indicates ±1SD of the spatial variability of values within each DBO site.

While linear trends can give an overall sense of change of environmental variables, they are not able to highlight interannual variability that is also a keystone feature of this region. As such, insights can be identified by displaying the actual time series data of annual sea ice persistence, timing of sea ice breakup/formation, and annual primary productivity as well (Fig 13). For those particular years that show relatively low annual persistence of sea ice, annual primary productivity is correspondingly high (and vice versa). It is worth noting that DBO3 is the only site that shows significant trends for all variables shown (timing of sea ice breakup/formation, annual sea ice persistence, and annual primary productivity). Furthermore, given the outlying behavior of sea ice in the Bering Sea in 2018 and 2019 (Fig 1), the observation of concomitant increasing responses of annual primary productivity during those individual years (e.g., Fig 13a) is important for a first-order assessment of the importance of sea ice decline to primary productivity.

**Fig 14:**
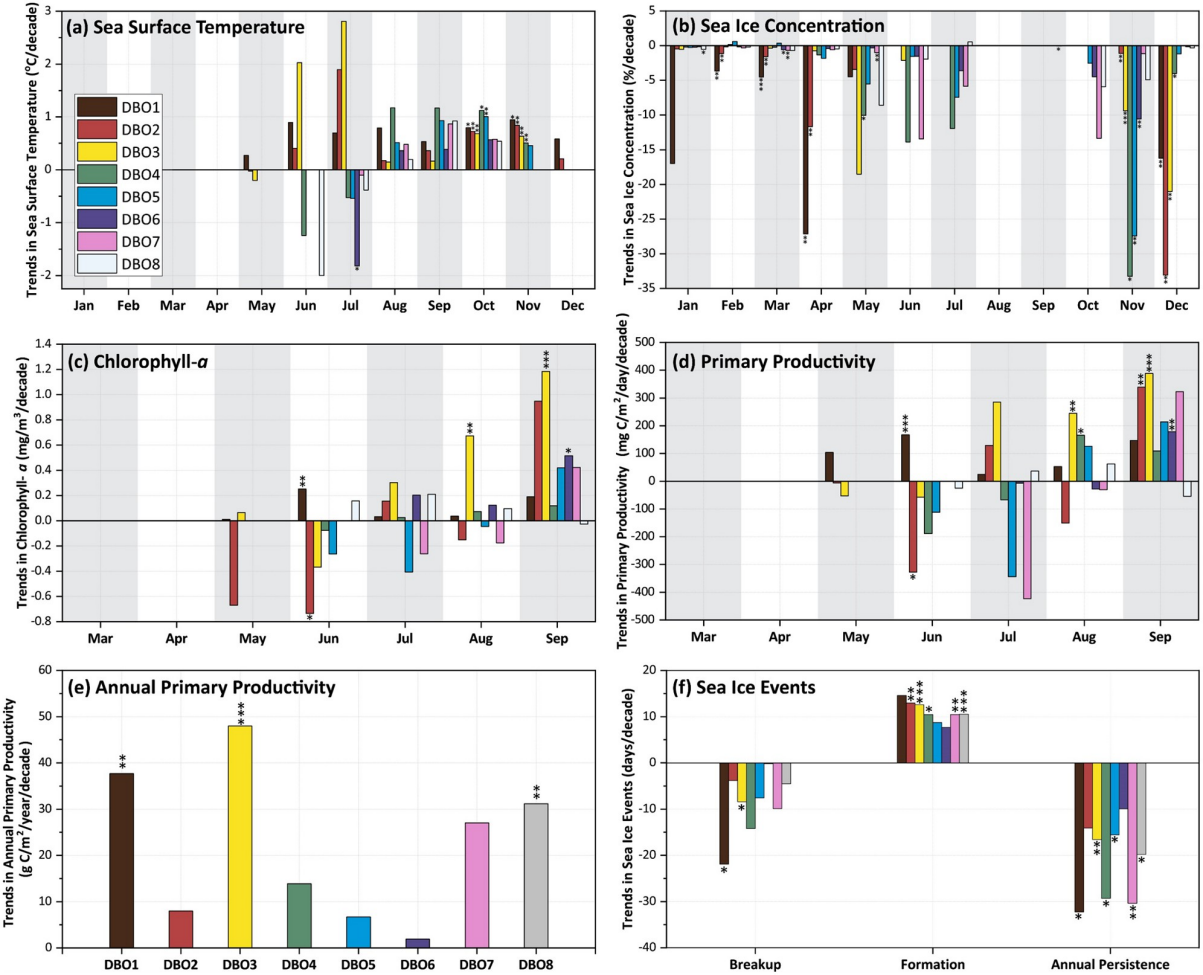
Summary plots of decadal trends (as indicated by the Theil-Sen median slope) in (a) sea surface temperature, (b) sea ice concentration, (c) chlorophyll-*a*, (d) primary productivity, (e) annual primary productivity, and (f) sea ice events (timing of sea ice breakup/formation and annual sea ice persistence). Statistical significance (based on a Mann-Kendall test for trend) is indicated by * (*p* < 0.1), ** (*p* < 0.05), or *** (*p* < 0.01).

While we can summarize the overall trends for each of the eight DBO sites, it is important to note that the spatial patterns in statistically significant trends are such that the DBO sites are not necessarily representative of the regions of greatest change in SST, sea ice, chlorophyll-*a*, and primary productivity. Nevertheless, it is important to quantify and summarize trends for all variables for the eight DBO sites (Fig 14). While trends in SST show the greatest increases at DBO3 (2.0°C/decade in June and 2.8°C/decade in July), these shifts are not statistically significant, based upon the analyses chosen. The most consistent, statistically significant trends in SST occur during the autumn, including during October (DBO1 = 0.79°C/decade, DBO2 = 0.73°C/decade, DBO3 = 0.68°C/decade, DBO4 = 1.1°C/decade, and DBO5 = 1.0°C/decade) and during November (DBO1 = 0.95°C/decade, DBO2 = 0.84°C/decade, DBO3 = 0.64°C/decade, and DBO4 = 0.5°C/decade). The only statistically significant cooling trend is observed at DBO6 during July (-1.8°C/decade). For sea ice concentrations, all significant trends are negative, with November exhibiting significant decreases for the most DBO sites (DBO2 = -1.1%/decade, DBO3 = -9.4%/decade, DBO4 = -33.3%/decade, DBO5 = -27.4%/decade, and DBO6 = -10.6%/decade). December also exhibits significant decreasing trends for DBO1-DBO4. DBO1 and DBO2 have also had significant decreases in sea ice concentrations across multiple winter/early spring months (February, March, April). Otherwise, significant trends in sea ice concentrations are spotty across DBO sites and months. Trends for chlorophyll-*a* and primary productivity track similarly to one another, although primary productivity increases are more statistically significant than those for chlorophyll-*a*. For chlorophyll-*a*, significant positive trends occur in June (DBO1 = 0.25 mg/m^3^/decade), August (DBO3 = 0.67 mg/m^3^/decade), September (DBO3 = 1.2 mg/m^3^/decade and DBO6 = 0.51 mg/m^3^/decade). Significant negative trends occur for DBO2 during June (-0.73 mg/m^3^/decade). For primary productivity, significant positive trends occur for June (DBO1 = 167 mg/m^2^/day/decade), August (DBO3 = 243 mg/m^2^/day/decade and DBO4 = 167 mg/m^2^/day/decade), and September (DBO2 = 338 mg/m^2^/day/decade, DBO3 = 402 mg/m^2^/day/decade, and DBO6 = 159 mg/m^2^/day/decade). Significant negative trends in primary productivity occur for DBO2 during June (-354 mg/m^2^/day/decade). Trends in annual primary productivity are significant at DBO1 (37.7 g C/m^2^/year/decade), DBO3 (48.0 g C/m^2^/year/decade), and DBO8 (31.2 g C/m^2^/year/decade). Lastly for sea ice events, breakup trends are significant at DBO1 (-21.9 days/decade) and DBO3 (-8.4 days/decade). More trends in the timing of seasonal sea ice formation are significant, including at DBO2 (12.9 days/decade), DBO3 (12.6 days/decade), DBO4 (10.5 days/decade), DBO7 (10.5 days/decade), and DBO8 (10.6 days/decade). Trends in annual sea ice persistence are significant at DBO1 (-32.3 days/decade), DBO3 (-16.6 days/decade), DBO4 (-29.3 days/decade), DBO5 (-15.6 days/decade), DBO7 (-30.4 days/decade), and DBO8 (-19.8 days/decade).

### Open water-primary productivity relationships

**Fig 15:**
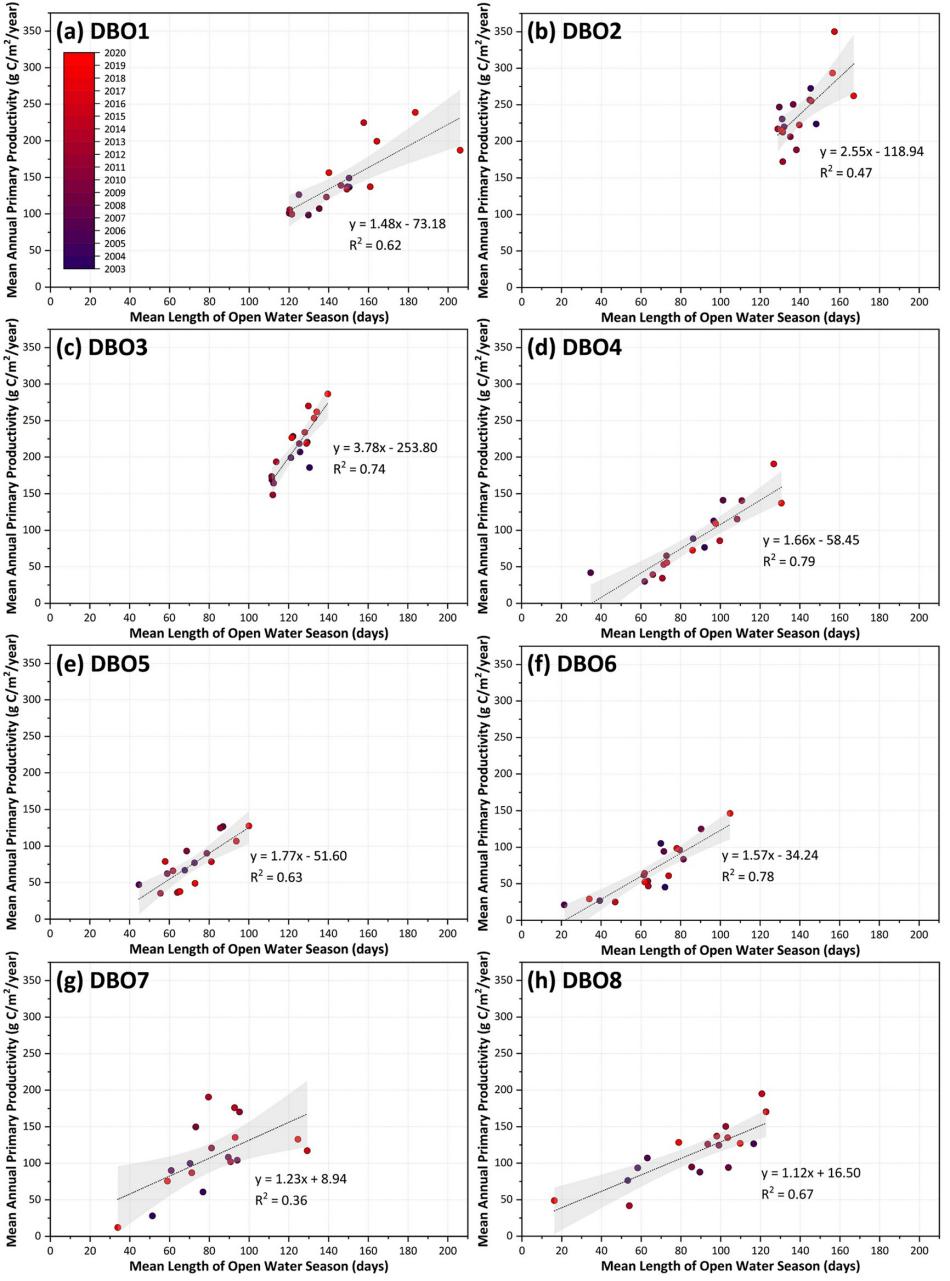
Relationships between length of open water season (March-September) and annual primary productivity (March-September) for each of the eight DBO sites. Linear regressions with 95% confidence intervals (shaded regions) are shown, with the year of each datapoint color coded using the color scale shown in (a).

**Fig 16:**
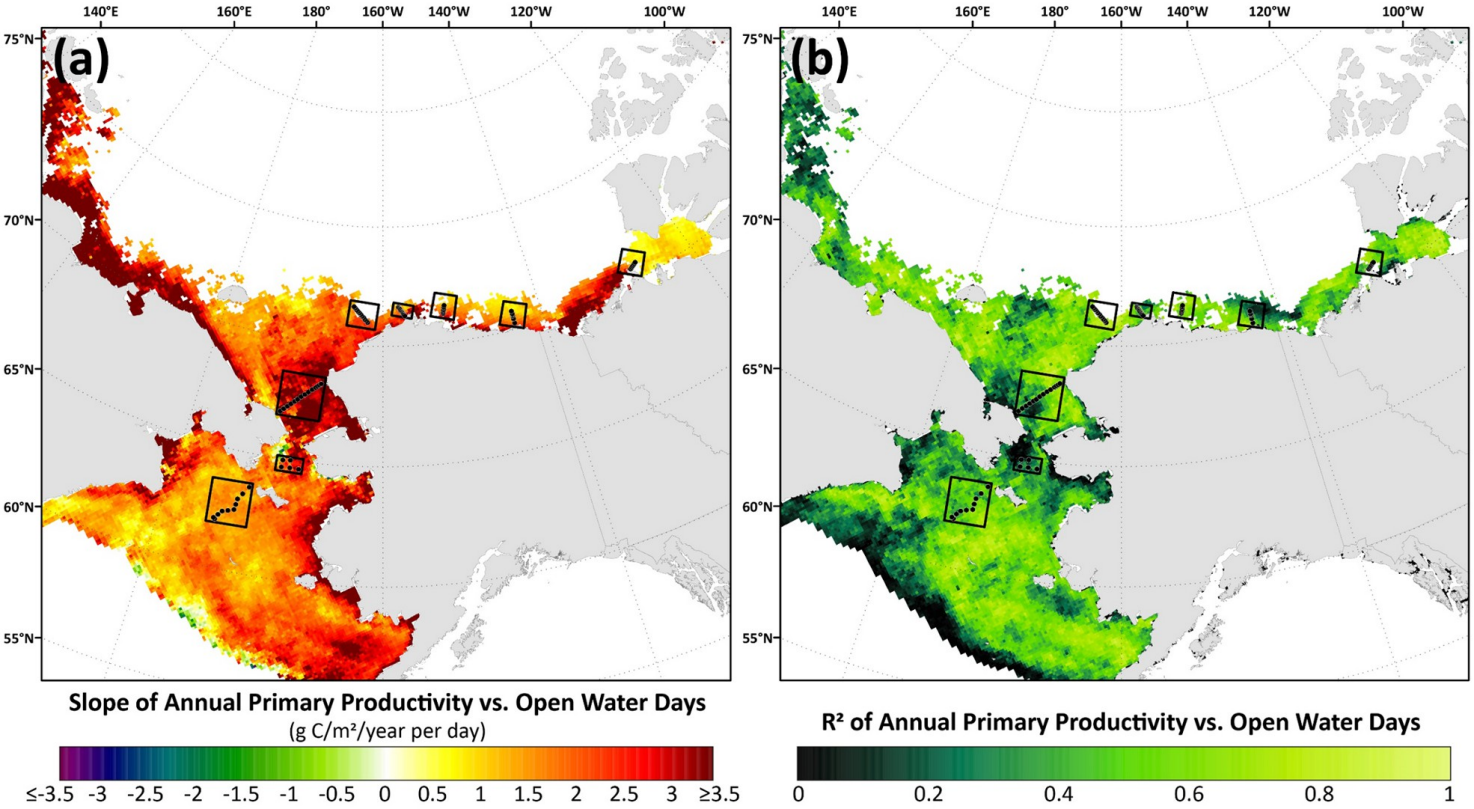
Results from the linear regression of annual primary productivity (March-September) and number of open water days (March-September) over the years 2003–2020 across the DBO sites, including both (a) slope and (b) R^2^. Basemap datasets from Natural Earth [49] and ESRI [50].

We investigated the strength of the relationships between annual primary productivity and open water days (March-September) for both the individual eight DBO sites (Fig 15) and spatially across the Pacific Arctic region (Fig 16) using simple linear regressions. Doing so for the individual DBO sites additionally allows direct observation of the interannual variability in both primary productivity and length of the open water season over the 2003–2020 time period. As such, the interannual range of the seasonal open water length (March-September) (i.e., the difference between the longest open water season and the shortest open water season over the 2003–2020 time period for a given DBO site) is highest at DBO8 (106.4 days), DBO4 (96.0 days), and DBO7 (95.2 days). This same calculated range is lowest for DBO3 (28.2 days) and DBO2 (38.1 days), demonstrating much less interannual variability in the length of ice cover at these sites. The same calculations can be made for annual primary productivity, where the greatest interannual ranges are at DBO2 (178.0 g C/m^2^/year) and DBO7 (178.5 g C/m^2^/year), and the smallest interannual ranges are at DBO5 (92.3 g C/m^2^/year) and DBO6 (124.9 g C/m^2^/year). As a result of the linear regressions, we see that the highest R^2^ values indicate that ~79% of the variance in mean annual primary productivity can be explained by the length of the open water season at DBO4, 78% for DBO6, and 74% for DBO3. DBO sites where open water explained the least of the variance in annual primary productivity include DBO7 (36%) and DBO2 (47%). In addition, the steepest slope of the primary productivity-open water relationship is found at DBO3 (3.8 g C/m^2^/year per day), meaning that for every day gained of the annual open water season (over the March-September period), primary productivity increases 3.8 g C/m^2^/year. The spatial representation of slope and R^2^ also provides important context for this primary productivity-open water relationship across the Pacific Arctic region (Fig 16). We observe the steepest slopes (≥3.5 g C/m^2^/year per day) across the study region in the southern Chukchi Sea just north of Bering Strait in the vicinity of DBO3 (Fig 16a). All slopes relating primary productivity to open water are positive except along the sea ice edge in the Bering Sea and in the western Bering Strait. The ability for open water days to explain the variance in primary productivity (as assessed by R^2^ values) is strong across the region (Fig 16b), with most values >0.6 for much of the Bering, Chukchi, and Beaufort Seas with many areas exhibiting R^2^>0.8. The lowest R^2^ values (<0.2) seem to congregate along the sea ice edge in the northern Bering Sea as well as in the western Bering Strait. This western Bering Strait region of low R^2^ values (encompassing portions of both DBO2 and DBO3) geographically coincides with some of the negative primary productivity-open water slopes (Fig 16a) as well as the statistically significant negative trends in June chlorophyll-*a* (Fig 9b) and June primary productivity (Fig 10b).

## Discussion and conclusions

This study provides a first concurrent assessment of satellite-based environmental variables (SST, sea ice, chlorophyll-*a*, primary productivity, and PAR) for all eight DBO sites across the Pacific Arctic region. While the DBO sites are often described as distributed across a latitudinal (or climate) gradient, there are exceptions to this general notion. In particular, although there is an expected clear cooling pattern with longer seasonal sea ice cover moving from DBO1 in the south to DBO6 in the north, DBO7 and DBO8 show warmer temperatures and less sea ice cover (compared to DBO6), which is likely associated with the warming effects of the Beaufort Gyre [75] and Mackenzie River outflows [76]. These broad-scale patterns are also reflected in the phenology of chlorophyll-*a* and primary productivity across the DBO sites, however these parameters have more complex variability because they are also affected by other factors such as nutrient availability, length of daylight, and grazing. In general, DBO2 and DBO3 have markedly higher mean annual primary production than any other DBO site (238.7 g C/m^2^/year and 214.5 g C/m^2^/year, respectively), which is in large part sustained by nutrient-rich waters along the western portion of the Bering Strait region [77]. These high concentrations of nutrients (maintained through high inputs as well as a well-mixed water column) can also be seen as surface nitrate concentrations remaining elevated at DBO2 even through the summer months, as most other sites experience depletion of nutrients to undetectable levels during July and August (Fig 5b). In contrast to the DBO sites of highest primary production, DBO4, DBO5, and DBO6 show the lowest rates of annual primary production and DBO1, DBO7, and DBO8 show middle-range rates of annual primary production (Fig 5d). Although DBO1 experiences the warmest SSTs and longest seasons of open water, total annual production is not higher here because nutrient availability plummets after May when the primary spring bloom depletes those nutrients that had been available earlier in the season [78]. While nutrient availability for DBO1 again increases later in the autumn (beginning in September) (e.g., [79]) (Fig 5b), neither chlorophyll-*a* concentrations nor primary production returns to the levels observed during spring (Fig 5e and 5f).

The most significant and consistent synoptic trends for the DBO sites have been those during late summer and autumn (warming SST during October/November, later shifts in the timing of sea ice formation, and increases in chlorophyll-*a*/primary productivity during August and September). However, this does not necessarily mean that important changes have not also occurred earlier in the season during spring. In fact, warming SSTs (Fig 6), declining sea ice cover (Fig 7), sea ice breakup becoming earlier (Fig 8), and shifts in chlorophyll-*a*/primary productivity (Figs 9 and 10) have additionally occurred during spring months across the region. These springtime trends, however, distribute in complex geographic patterns and do not always align with DBO sites specifically. For instance, some of the steepest observed declines in sea ice have occurred during winter and early spring months (January–May) in the northern Bering Sea in the vicinity of DBO1 (Fig 7a–7e), with these patterns driven primarily by later sea ice formation and earlier sea ice breakup in the last several years (particularly since 2015) with unprecedented sea ice lows during winters 2017–2018 and 2018–2019 owing primarily to warm, southerly winds in February/March [8]. For instance, during winter 2017–2018, DBO1 experienced only 17 days of sea ice cover (days with >15% sea ice concentration), compared to a long-term mean (1981–2010) of 149 days per year (Fig 13a). Some of the ecosystem consequences of these early sea ice breakups and extreme lows in sea ice cover have been (perhaps counterintuitively) later phytoplankton blooms during June rather than in May as shown using the same data in a previous report [9]. This is supportive of the Oscillating Control Hypothesis [80] where early, ice-associated blooms in cold water occur during years of late ice retreat, whereas later, open-water blooms occur in warm water during years of early ice retreat (i.e., before mid-March). Indeed, we observe significant increases in chlorophyll-*a* and primary production in June at DBO1 (Figs 9b and 10b) (driven largely by increases during June 2018 and 2019) with pockets of both increases and decreases (yet not statistically significant) trends in May (Figs 9a and 10a). Despite the seasonal heterogeneity of chlorophyll-*a*/primary productivity trends at DBO1, this site is one of only three DBO sites where a significant overall increase in annual productivity over the 2003–2020 time period (Fig 14e) has been observed, which is primarily contributed to by both increases during June and late season increases during September (Figs 10b, 10e and 13d). Trends in PAR across months (Fig 12) do not appear coincident with significant trends in primary productivity (Fig 10) at any of the DBO sites, except for DBO1 during June when increases in PAR (Fig 12d) may be a contributing factor for increases in primary productivity (Fig 10d).

Additional heterogenous trends within DBO sites are highly apparent for sites DBO2 and DBO3. DBO2 is the only site with significant negative trends in chlorophyll-*a* and primary productivity over 2003–2020, which in both cases occurred during the month of June (Fig 13c and 13d). On closer inspection of the spatial trends during June, there is a distinct region of statistically significant negative trends in both chlorophyll-*a* (Fig 9b) and primary productivity (Fig 10b) in the western Bering Strait region that is otherwise typically associated with relatively high nutrient/high productivity waters [77] (S4 and S5 Figs). This region of decreasing chlorophyll-*a*/primary productivity that encompasses nearly all of DBO2 (and also the western half of DBO3) does not seem associated with cooling trends of SST (Fig 6f) or shifts in sea ice (Fig 7f), so we hypothesize that there may be some ongoing shifts in nutrient delivery via the deep Pacific Anadyr waters that typically deliver high nutrient waters through the Bering Strait across the western Chukchi Sea shelf as winter water currents [81,82]. There may be some precursory evidence of this during May, when we observe significant decreasing trends in chlorophyll-*a* (Fig 9a) and primary productivity (Fig 10a) farther south (i.e., upstream of Bering Strait) in the Gulf of Anadyr with no concurrent significant trends in sea ice cover or SST. A recently published satellite- and modeling-based study provides a more in-depth analysis of these declining primary productivity trends during June in the western Bering Strait [83]. While these patterns manifest as clear negative trends for DBO2 during June, they impart a more complex story for DBO3 because they only cover the western half of this DBO site. During June, the western half of DBO3 exhibits these significant negative trends in chlorophyll-*a* (Fig 9b) and primary productivity (Fig 10b), yet significant positive trends are observed in the eastern half in North American waters. As a result of these bifurcated trends, there are no overall significant June trends for DBO3 (Fig 13c and 13d). Nonetheless, trends in annual primary productivity are highest at DBO3 compared to all other DBO sites at 48.0 g C/m^2^/year/decade (Fig 14e), primarily owing to increasing contributions later in the season (August and September) (Fig 14d) that may be driven by well-mixed nutrient-rich winter water during these late season months [84].

As highlighted previously, the most significant and synoptic trends for the DBO sites have been those during late summer and autumn (warming SST during October/November, later shifts in the timing of sea ice formation, and increases in chlorophyll-*a*/primary productivity during August and September). Sea ice variability is controlled by multiple drivers, including cooling or warming air temperatures, changes in atmospheric circulation and ice motion, shifts in cloud cover, and advected ocean heat [85]. Preconditioning of sea ice and ice-albedo feedbacks can also set the stage for even more dramatic changes once shifts in sea ice are initiated, where earlier sea ice retreat allows for more solar radiation to be absorbed by ocean waters, resulting in seawater warming and an amplified delay in sea ice formation the following autumn [86]. These shifts in the later timing of sea ice formation are ubiquitous across the DBO sites (Fig 8c), which is also associated with geographically widespread significant warming SST trends during October and November (Fig 7j and 7k). These longer open water seasons may allow for increased winds, particularly during autumn (resulting from reduced atmospheric stability as a consequence of increased temperature and turbulent fluxes [87]), which in turn causes vertical mixing of nutrients that instigates a secondary autumn bloom [15,79]. However, this may be countered to a certain degree by enhanced stratification through expected increases in freshwater input from melting ice and river runoff [88].

While an increased supply of nutrients has been shown to be a dominant driver of increased primary productivity in recent years across the Arctic Ocean [17], our study shows that low light availability owing to sea ice presence still appears to play a primary limitation for production across many of the DBO sites. In particular, the number of open water days explains the variance of annual primary production at sites DBO3, DBO4, and DBO6 to the greatest extent, with DBO3 showing the greatest change in productivity (3.8 g C/m^2^/year) for every day of open water added to the season. This may indicate that nutrients are sufficiently available at these sites and that low light availability owing to sea ice presence still remains a primary limitation of annual production. However, with the expected continuation of sea ice decline and a lengthening open water season throughout the Pacific Arctic region (as well as shifts from multiyear to first-year sea ice that can affect the under-ice light field and ice/under-ice algal biomass alongside open water phytoplankton), the primary limitations on production may continue to shift. While patterns in sea ice and primary productivity for 2021 and 2022 have been spatially heterogenous across the Pacific Arctic region [89,90], neither of these recent years exhibited the anomalously low sea ice cover observed in the Bering Sea during winters 2017–2018 and 2018–2019 (e.g., [8,9]). Nevertheless, these recent extreme conditions have indeed provided insight into how primary productivity may respond to continued sea ice declines. As such, it will be critical to continue monitoring environmental variables across the DBO, both via satellite and in situ observations, as we lengthen our time series to better understand how these globally significant ecosystems (including not only primary productivity but also phytoplankton community and size structure) will be impacted by ongoing and future climate warming.

## Supporting information

S1 File (PDF)
