# Supplementary material for: A comprehensive satellite-based assessment across the Pacific Arctic Distributed Biological Observatory shows widespread late-season sea surface warming and sea ice declines with significant influences on primary productivity
Source: PLoS One. 2023 Jul 11;18(7):e0287960. doi: 10.1371/journal.pone.0287960 (PMC10335666; doi:10.1371/journal.pone.0287960)
Supplement: S1 File — (PDF) [file pone.0287960.s001.pdf]

# A comprehensive satellite-based assessment across the Pacific Arctic Distributed Biological Observatory shows widespread late-season sea surface warming and sea ice declines with significant influences on primary productivity

Karen E. Frey[1*], Josefino C. Comiso[2], Larry V. Stock[2], Luisa N. C. Young[1], Lee W. Cooper[3] & Jacqueline M. Grebmeier[3]

[1]Graduate School of Geography, Clark University, Worcester, Massachusetts, USA
[2]Cryospheric Sciences Laboratory, NASA Goddard Space Flight Center, Greenbelt, MD, USA
[3]Chesapeake Biological Laboratory, University of Maryland Center for Environmental Science, Solomons, MD, USA

* Corresponding author
Email: kfrey@clarku.edu (KEF)

**Supporting Information:**

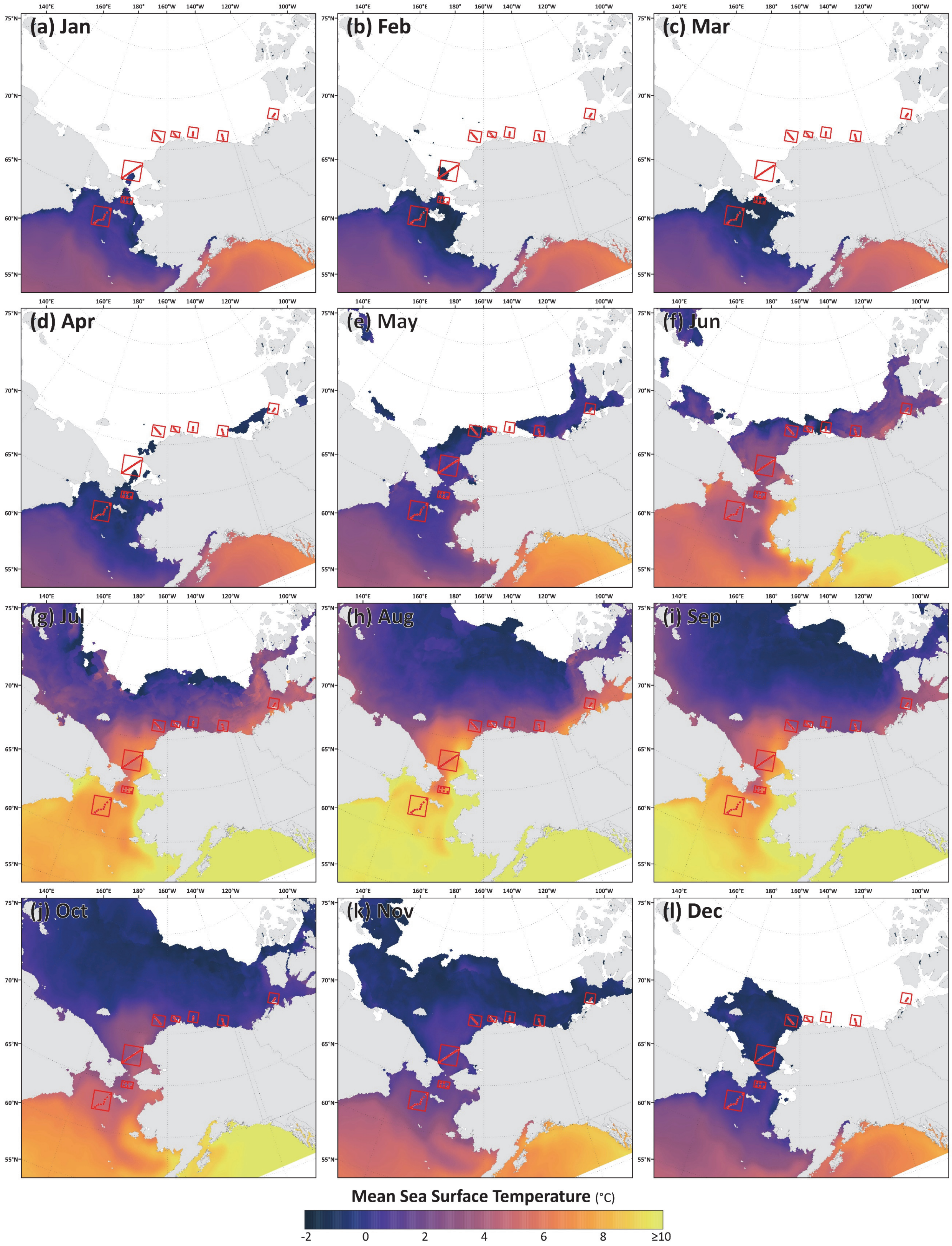


**Figure S1.** Satellite-derived monthly mean values of sea surface temperature (SST) across the Pacific Arctic region over the 2003–2020 period.

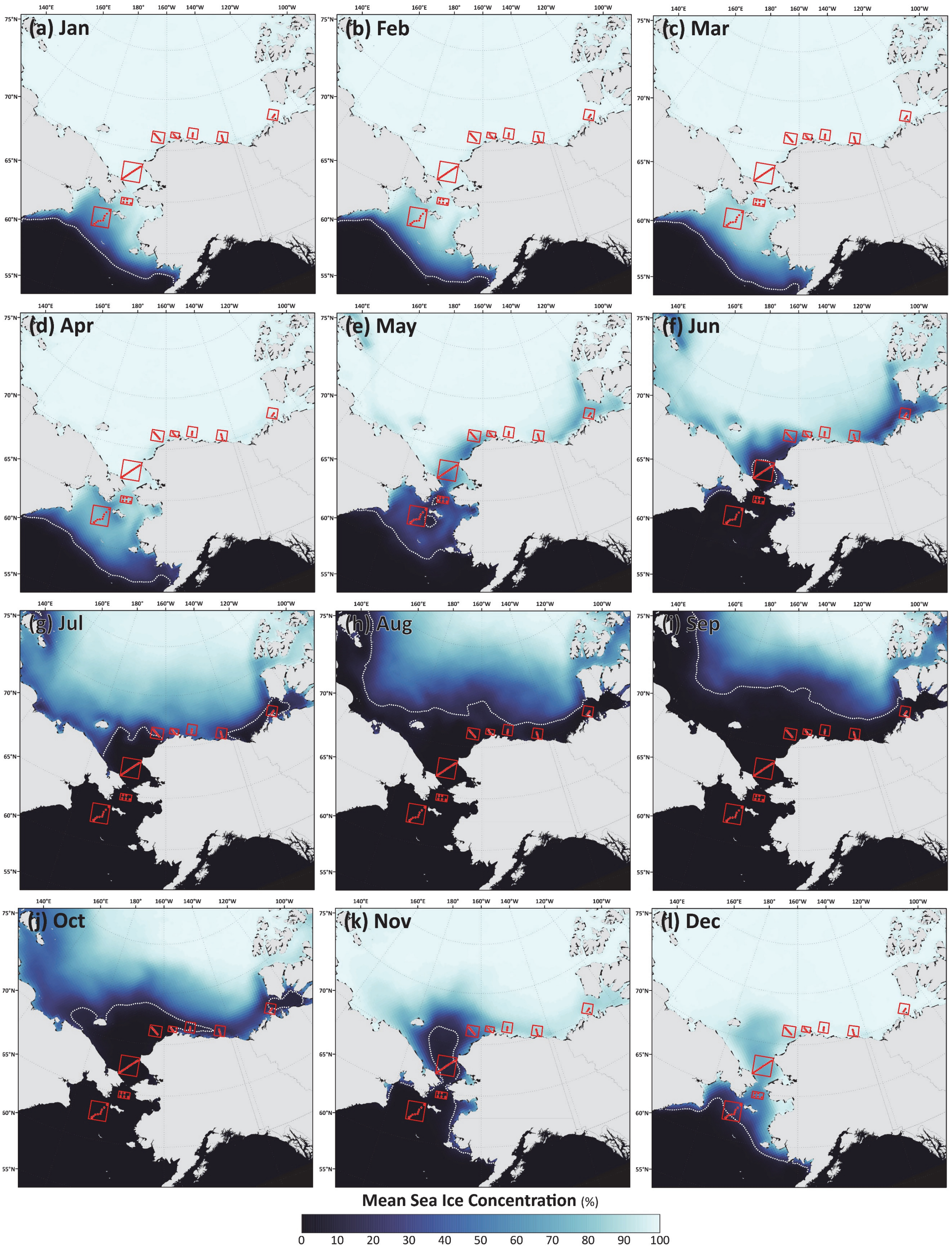


**Figure S2.** Satellite-derived monthly mean values of sea ice concentration across the Pacific Arctic region over the 2003–2020 period. White dotted contours indicate mean 15% sea ice concentration.

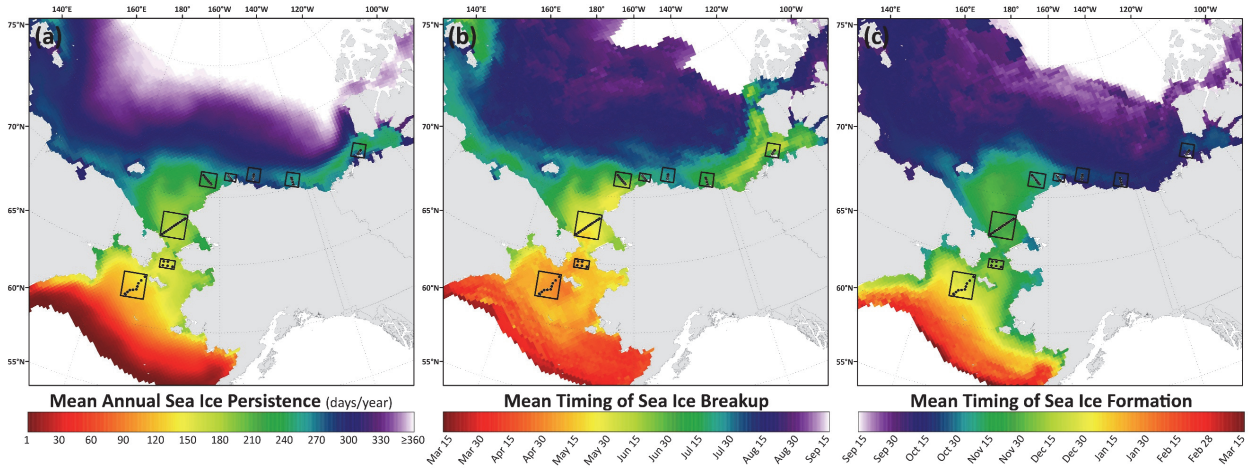


**Figure S3.** Satellite-derived mean values across the Pacific Arctic region for (a) annual sea ice persistence (i.e., number of days per year with sea ice cover), (b) timing of sea ice breakup, and (c) timing of sea ice formation over the 2003–2020 period. White areas represent no ice (in the south) or areas of multi-year ice (in the north) that did not experience seasonal sea ice breakup or formation.

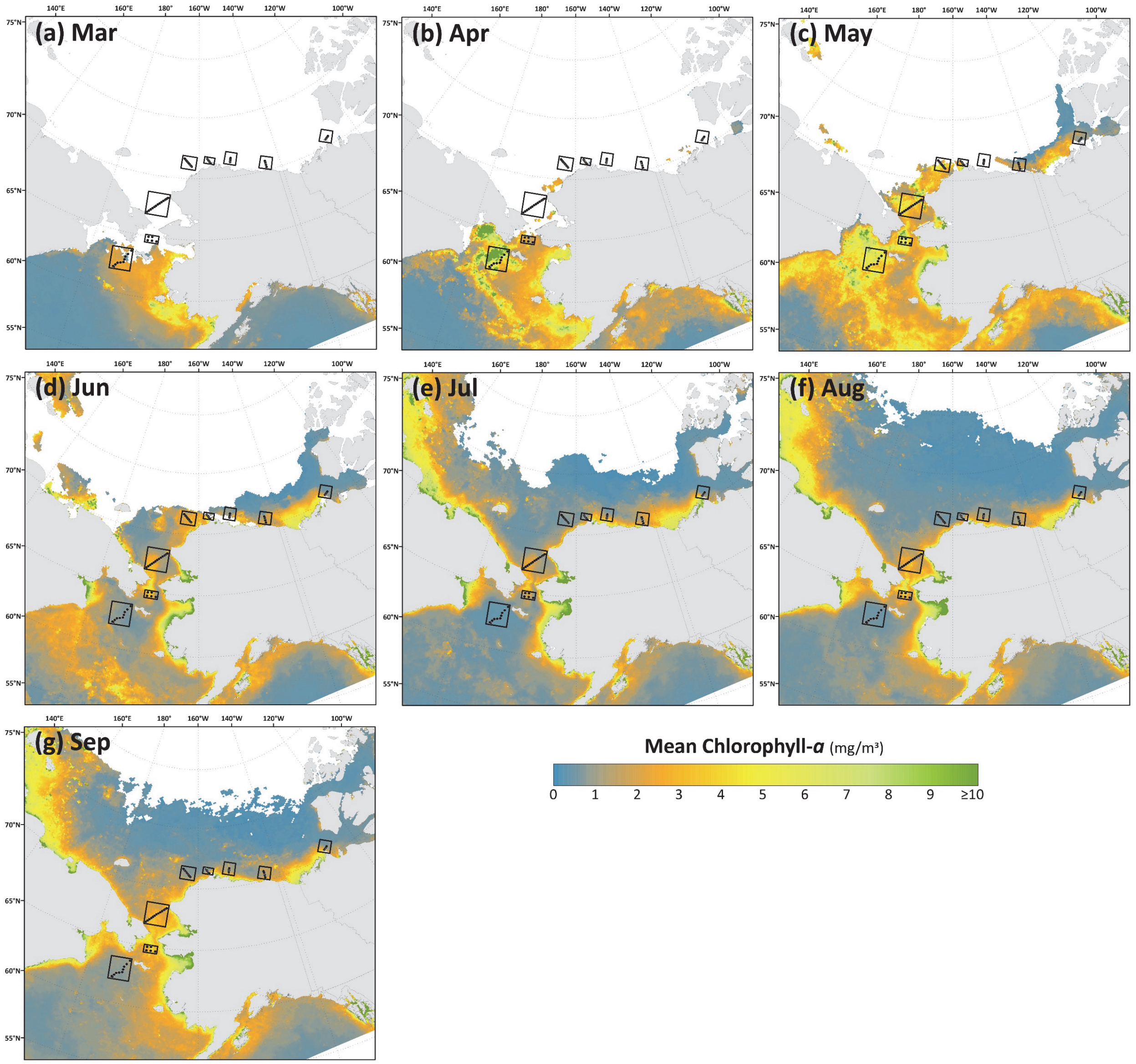


**Figure S4.** Satellite-derived monthly mean values of chlorophyll-*a* concentrations across the Pacific Arctic region over the 2003–2020 period.

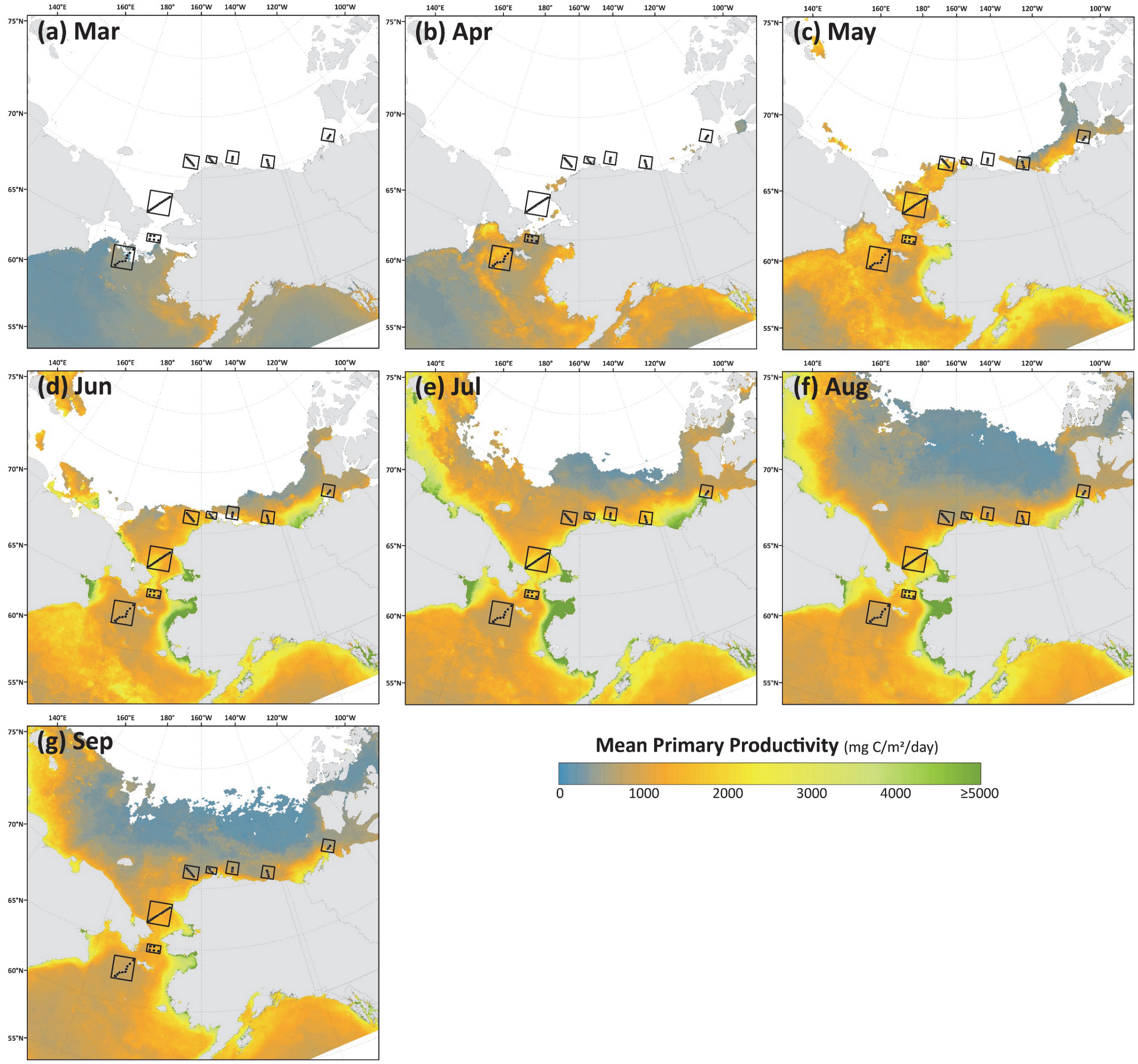


**Figure S5.** Satellite-derived monthly mean values of primary productivity across the Pacific Arctic region over the 2003–2020 period.

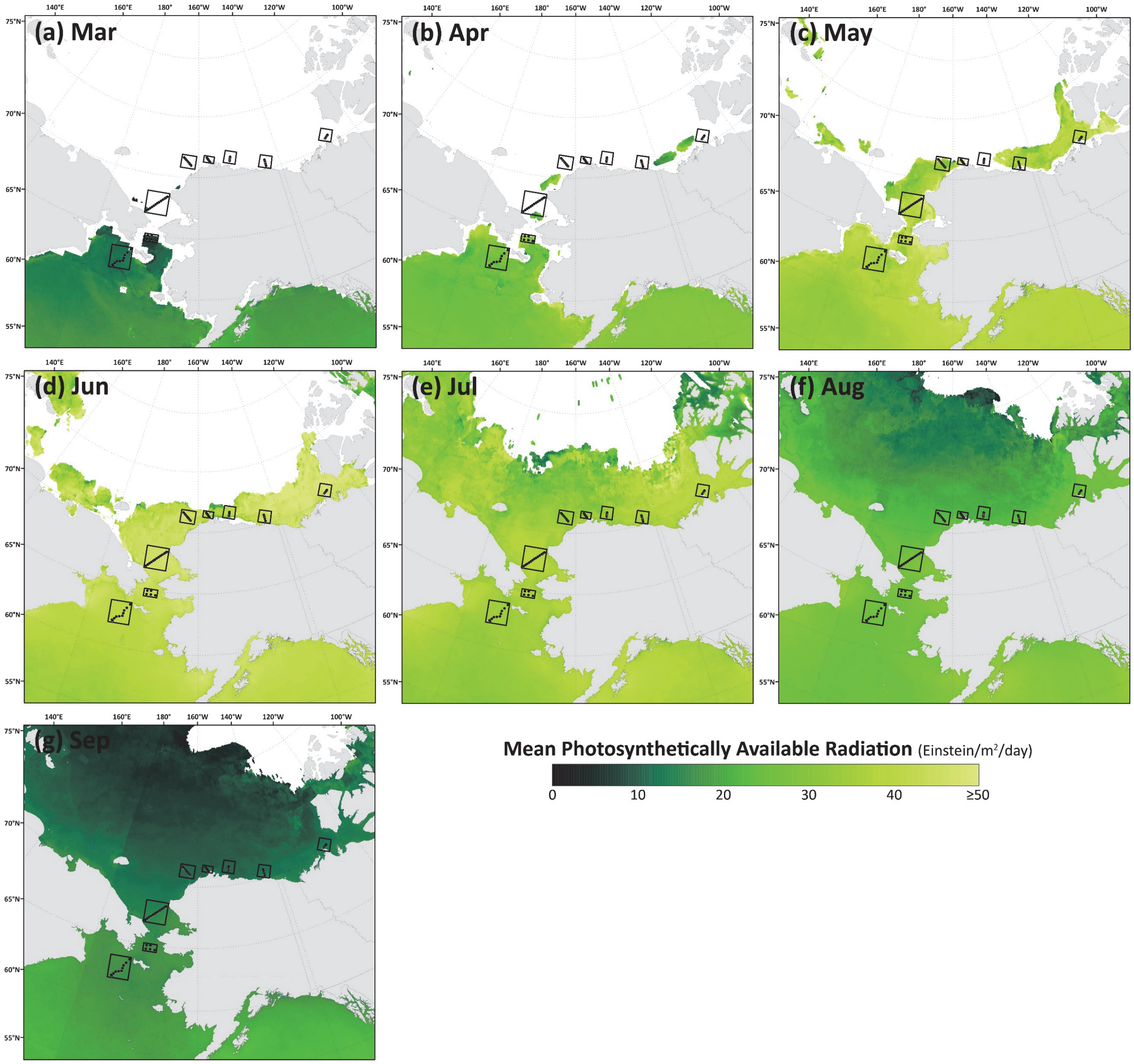


**Figure S6.** Satellite-derived monthly mean values of photosynthetically available radiation across the Pacific Arctic region over the 2003–2020 period.
